# Nanoreporter for Real‐Time Monitoring of Inflammasome Activity and Targeted Therapy

**DOI:** 10.1002/advs.202204900

**Published:** 2023-01-05

**Authors:** Dipika Nandi, James Forster, Anujan Ramesh, Anh Nguyen, Hariharan Bharadwaj, Ashish Kulkarni

**Affiliations:** ^1^ Department of Chemical Engineering University of Massachusetts Amherst MA 01003 USA; ^2^ Department of Veterinary and Animal Sciences University of Massachusetts Amherst MA 01003 USA; ^3^ Department of Biomedical Engineering University of Massachusetts Amherst MA 01003 USA; ^4^ Center for Bioactive Delivery Institute for Applied Life Sciences University of Massachusetts Amherst MA 01003 USA; ^5^ Department of Pathology UMass Chan Medical School‐Baystate Springfield MA 01107 USA

**Keywords:** Caspase‐1, diagnostics, gasdermin‐D, gouty‐arthritis, inflammasome, theranostics

## Abstract

Inflammasome activation is associated with a myriad of inflammatory diseases. However, existing methods provides a limited understanding of spatiotemporal kinetics of inflammasome activation, with restricted scope for early detection of associated treatment efficacy. This limitation offers an opportunity for the development of biocompatible in‐vivo inflammasome monitoring tools with translational prospects. To achieve this, they report developing a pair of lipid‐based nanoparticle systems, a reporter nanoparticle consisting of a caspase‐1 activatable probe alone, and a theranostic nanoparticle combining the probe with an inflammasome‐inhibiting drug. This biocompatible platform enhances the probe's residence time in circulation by preventing its opsonization and allowing its sustained release over time. Their results demonstrate the specificity of reporter nanoparticles towards caspase‐1 activity and provides early‐on monitoring of inflammasome activation both in‐vitro as well as in‐vivo. Additionally, the delivery of disulfiram, an inflammasome‐inhibiting drug, along with reporter probe using theranostic nanoparticles enables real‐time tracking of treatment efficacy in the gouty‐arthritis inflammatory model. In summary, they report an unparalleled pair of the inflammasome‐associated reporter and theranostic platforms suited not only for diagnostic applications but can also detect inflammasome‐targeted treatment efficiency in real‐time. These findings establish two novel, sensitive nanotools for non‐invasive evaluation of inflammasome‐targeted immunotherapy.

## Graphical abstract

Monitoring the kinetics of Inflammasome activation could offer insights into their role in disease progression. A reporter nanoparticle consisting of a caspase‐1 activatable probe and a theranostic nanoparticle combining the activatable probe with an inflammasome‐inhibiting drug is developed. The reporter nanoparticle provided early‐on monitoring of inflammasome activation, and theranostic nanoparticle enabled real‐time tracking of treatment efficacy in gouty‐arthritis inflammatory model.

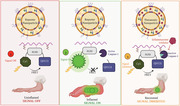

## 1 Introduction

Inflammasome activation in different tissues is known to be associated with several chronic and acute inflammatory diseases.^[^ ^1^, ^2^ ^]^ This form of innate immune response involves the assembly of three major proteins popularly known as the sensor, adaptor, and inactive enzyme, into a multimeric protein complex, which subsequently results in proximity‐induced activation of the caspase‐1 enzyme.^[^ ^3^, ^4^ ^]^ The activated enzyme further cleaves the inactive downstream proteins (including cytokines and pore‐forming protein, gasdermin‐D) to their respective active forms, eventually leading to a form of cell death termed pyroptosis, which is associated with the release of inflammatory cytokines, IL‐1*β* and IL‐18 resulting in enhanced immune response.^[^ ^5^ ^]^ The first participant of this molecular assembly is a cytosolic receptor, which is responsible for sensing pathogenic or sterile insults leading to foreign invasion or tissue damage. Upon threat sensing, these receptors undergo oligomerization and conformational change in order to make their pyrin domains available for binding to the second participant known as ASC (apoptosis‐associated speck‐like protein containing caspase activation and recruitment domain) adaptor protein. ASC forms discrete foci or speck like structures of around 1 µm via molecular assembly, which have been extensively utilized to visualize inflammasome assembly until now.^[^ ^6^ ^]^ This is followed by recruitment of zymogen procaspase‐1 to the site of ASC‐threat sensor complex in order to facilitate its proximity induced autocatalytic cleavage to generate active caspase‐1 which further results in downstream signaling.

Several attempts have been made in the past to develop in vitro and in vivo monitoring tools for individual inflammasome components by employing fluorescence, bioluminescence, near‐infrared and radiolabeling based approaches, which helped in better understanding of the signaling cascade.^[^ ^7^ ^]^ However, only a few studies have offered insights into the role of inflammasome activation in an in vivo setting during a disease progression.^[^ ^8^, ^9^, ^10^, ^11^ ^]^ Furthermore, there are no studies on systems to test the associated treatment efficacy noninvasively in real‐time, which provides an opportunity for the development of new visualization tools for inflammasome sensing. Some of the recent in vivo techniques include fluorescent reporter mice such as bone marrow (BM)‐chimeric ASC‐GFP retrogenic model and ASC‐citrine/Cre+ model.^[^ ^8^, ^12^ ^]^ Both these models provided a better understanding of the importance of ASC speck formation in inflammasome response and its role in regulation of downstream innate and adaptive immunity during in vivo settings. Few other transgenic reporter mice encompassed IL‐1R1^GR/GR^ (globally restored) and IL‐1*β* based dual operating luciferase (IDOL) models, that provided a better understanding of IL‐1R1 distribution and IL‐1*β* stimulation.^[^ ^12^, ^13^ ^]^ Even though all the above mentioned transgenic mouse models have served as great tools for dissecting the inflammasome signaling pathway, they require genetic modifications which render them inefficient for clinical applications. Few other detection platforms included biosensors specific to caspase‐1 or IL‐1*β*  (iGLUC) which provided information of one particular time point but failed to lay out the spatiotemporal kinetics.^[^ ^14^, ^15^, ^16^ ^]^ Recently, an NIR‐FRET based caspase‐1 probe was reported by Ko et. al. which helped in noninvasive real‐time imaging of inflammasome in various inflammatory diseases.^[^ ^9^ ^]^ However, this system has few shortcomings such as short circulating half‐life, reduced bioavailability and solubility which makes them inefficient for clinical use. All of these limitations can be overcome by the use of biocompatible lipid nanoparticles which not only increases the bioavailability of the probe in inflamed sites but also increases their retention in the circulation.^[^ ^17^, ^18^ ^]^ They also eliminate the need for genetic manipulations and can provide spatiotemporal kinetics for extended period of time due to sustained release of probe.^[^ ^18^, ^19^ ^]^ In compliance with this, we proposed to design a liposomal nanoreporter encapsulated with caspase‐1 cleavable probe, that could enable extended real‐time monitoring of inflammasome in vivo, during an inflammatory disease progression (Figure S1, Supporting Information). Furthermore, we realized that there are no clinically translatable platforms that can simultaneously track and treat NLRP3 mediated inflammation, this created a need for developing a platform capable of differentiating treatment responders in order to identify the right time and most efficient therapy in real‐time.^[^ ^7^ ^]^ In an attempt toward that, we chose to codeliver drug along with reporter probe to develop theranostic platform using similar liposomal nanoplatforms. This will ensure enhanced bioavailability and improved cellular retention of drug due to sustained release and facilitate its tracking at the same time.

Previous studies have shown a few correlation of nanomaterial properties with that of inflammasome activation and have reported some polymer and lipid nanomaterials that could activate inflammasome, but there have been no attempts to utilize this knowledge for developing efficient diagnostic and theranostic tools.^[^ ^20^, ^21^, ^22^, ^23^, ^24^ ^]^ Here for the first time, we have attempted to engineer an inflammasome nanoreporter utilizing the DOPC (1,2‐dioleoyl‐sn‐glycero‐3‐phosphocholine) nanoparticles which have been previously reported not to induce signal 2 and thus are safer to use for inflammasome immunotherapy.^[^ ^21^ ^]^ Additionally, for the first time an attempt has been made to develop inflammasome specific theranostic platform by combining the drug and the probe simultaneously in a single nanoplatform. Overall, we rationalized that nanoparticle delivering reporter probe specifically activatable by inflammatory caspases could enable real‐time monitoring of inflammation and associated therapeutics.

## 2 Results and Discussion

**Figure 1:**
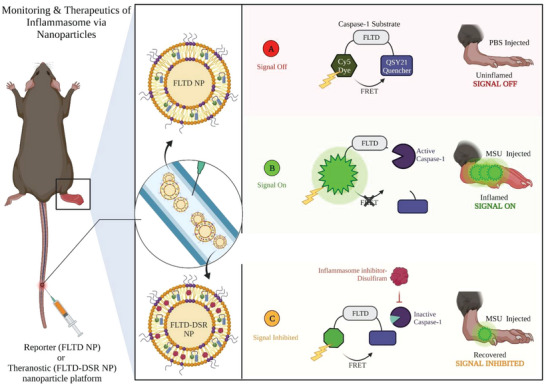
Schematics illustrating the activity of reporter and theranostic liposomal nanoparticles for inflammasome‐specific monitoring and therapy. Reporter nanoparticle (FLTD NP) are encapsulated with caspase‐1 cleavable imaging probe. This probe consists of Cy5 dye and QSY21 quencher conjugated via caspase‐1 substrate containing peptide sequence, K‐FLTD‐KC. a) FLTD NP generates no signal in uninflamed tissue as a result of intact caspase‐1 substrate bringing Cy5 and QSY21 in proximity less than 10 nm (FRET phenomenon), leading to quenching of Cy5 signal. b) FLTD NP generates a high signal to noise ratio in inflamed tissue due to presence of active caspase‐1 enzyme resulting from inflammasome assembly. This enzyme cleaves the ‐FLTD‐ substrate after “D” to generate sufficient distance between Cy5 dye and QSY21 quencher, greater than 10 nm, allowing the Cy5 to fluoresce. Theranostic nanoparticle (FLTD‐DSR NP) contains an additional inflammasome inhibiting drug, disulfiram, to monitor the treatment efficacy in real‐time. c) The reporter signal induced by FLTD DSR NP is indicative of therapy effectiveness. Disulfiram drug impedes inflammasome assembly and activation by inhibiting NFkB and gasdermin‐D. Inflammasome inhibition overall lowers the activation of caspase‐1 enzyme leading to reduced reporter signal in recovered tissue when compared with the inflamed tissue. The decrease in reporter signal is associated with treatment responsiveness.

In this study, we report a pair of inflammasome activatable nanoreporter and nanotheranostic platforms that not only enable monitoring of complex assembly but also help in tracking the related treatment efficacy. These liposomal nanoparticles are classified into two distinct nanoparticle platforms, a reporter nanoparticle and theranostic nanoparticle (**Figure** **1**), respectively. The reporter nanoparticle, known henceforth as FLTD NP, is encapsulated with only reporter probe, ‐FLTD‐, specifically cleavable by inflammasome‐associated caspase (caspase‐1) which can monitor its assembly as well as activation in real‐time during disease progression (Figures S1 and S3, Supporting Information).^[^ ^25^ ^]^ The designed FLTD peptide reporter mimics the caspase‐1 cleavable peptide sequence in the pore‐forming protein gasdermin‐D. Caspase‐1 is known to cleave gasdermin‐D at the P1 aspartate group (FLTD), as shown in Figure S2 (Supporting Information). The theranostic nanoparticle, henceforth known as FLTD‐DSR NP, helps in identifying the inflammasome related treatment efficacy in the real‐time due to the encapsulation of an inflammasome‐inhibiting drug, disulfiram (DSR),^[^ ^26^, ^27^, ^28^ ^]^ in addition to the ‐FLTD‐ probe. We rationalized that upon inflammation, FLTD NPs will result in a high signal to noise ratio, due to which in inflamed tissue we anticipate observing a significantly greater signal when compared to uninflamed tissue (Figure 1a,b). We also anticipate observing a downregulation in the reporter signal intensity when the treatment starts to work (Figure 1c), as a consequence of inflammasome‐inhibiting drug, disulfiram's activity. Reporter and theranostic nanoparticles were synthesized using self‐assembling of colipids DOPC (1,2‐dioleoyl‐sn‐glycero‐3‐phosphocholine) and DSPE‐PEG (2000)‐Amine (1,2‐distearoyl‐sn‐glycero‐3‐phosphoethanolamine‐N‐), encapsulated with only reporter probe (FLTD NPs) or an additional inflammasome‐inhibiting drug (FLTD‐DSR NPs). The reporter probe contains inflammatory caspase substrate found in gasdermin‐D, ‐FLTD‐, which is cleavable by all inflammatory caspases including caspase‐1^[^ ^29^ ^]^ and thus is an indicative of upregulation of inflammasome specific signaling. FLTD caspase substrate is conjugated to Cy5 NIR fluorescent dye and QSY21 quencher on either side leading to low background signal in native state due to FRET phenomenon (Figure S2, Supporting Information).

The inflammasome inhibiting drug paired with the reporter system in FLTD‐DSR NPs is disulfiram which inhibits NF*κ*B and pore‐forming protein gasdermin‐D. We chose to utilize disulfiram due to its high efficiency in inhibiting signal 1 via NF*κ*B inhibition^[^ ^30^, ^31^ ^]^ and signal 2 via inhibition of N‐terminal gasdermin‐D translocation to plasma membrane,^[^ ^26^ ^]^ which proves it as an efficient pair with gasdermin mimic caspase‐1 responsive probe. By using this pair, we expect to observe reduction in reporter signal due to do an overall downregulation in caspase‐1 cleavage and activity, subsequently alleviating pyroptosis. Previous literature has also shown its efficiency in various inflammatory disease in in vivo models which proves it to be suitable for proof‐of‐concept studies focused on identifying in vivo efficacy.^[^ ^26^, ^28^ ^]^ Similar to previous liposomal systems in our lab,^[^ ^17^, ^32^ ^]^ FLTD and FLTD‐DSR nanoparticles were synthesized employing the thin film hydration approach to encapsulate either just the imaging probe or an additional drug in the self‐assembling colipids backbone. Both FLTD and FLTD‐DSR NPs were found to be stable in storage as well as physiological conditions for an extended period of time with hydrodynamic diameter of around 200 nm, a neutral surface zeta potential, and an average PDI of 0.381. In vitro and in vivo nanoparticle activity studies revealed its efficient inflammasome‐associated diagnostic and theranostic capability to determine the inflammation intensity enabling a correlation with disease severity as well as treatment efficacy early‐on as corroborated by ex‐vivo studies. These studies, for the very first time, successfully validate the inflammasome centric diagnostics and therapy simultaneously in MSU‐induced gouty arthritis and proves to be a robust monitoring and treatment tool for inflammatory disease pathologies mediated by the NLRP3 inflammasome. Overall, they serve as a potential noninvasive platform for screening of different inflammasome immunotherapy efficacies in various disease conditions.

**Figure 2:**
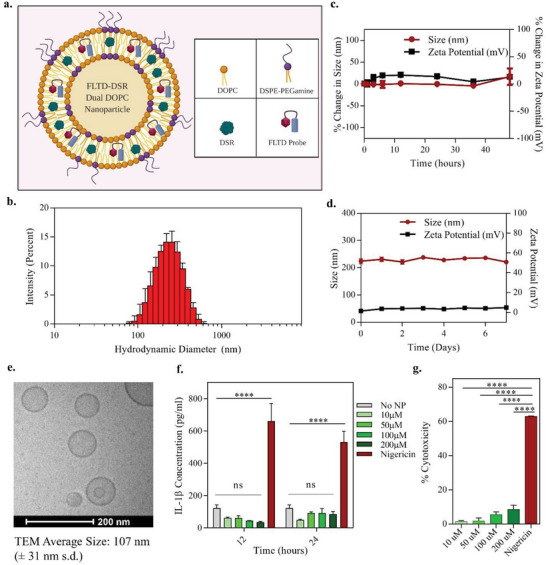
Theranostic FLTD‐DSR nanoparticle characterization. a) Schematic representation of theranostic nanoparticle components and self‐assembling. Theranostic nanoparticles, or FLTD‐DSR dual nanoparticle is synthesized by physically encapsulating caspase‐1 responsive FLTD probe and inflammasome inhibiting disulfiram drug in self‐assembling colipid backbone facilitated by DOPC and DSPE‐PEG‐amine. b) DLS intensity plot for FLTD‐DSR NPs showing an average hydrodynamic diameter of 228 nm. Data represents mean ± s.d. c) Graph displays the percentage change in size and zeta potential of FLTD‐DSR NPs incubated in human serum for a total duration of 48 h. d) PBS Stability plot shows size and zeta potential of FLTD‐DSR NPs in PBS over a period of 7 days. Data shown in c and d are mean ± s.e.m. (*n* = 3). e) Cryo‐TEM image of FLTD‐DSR NPs displaying an average size of 107 nm (± 31 nm s.d.), with all but one nanoparticle exhibiting the expected unilamellar morphology. f) IL‐1b release in supernatant of iBMDMs treated with different concentrations of blank nanoparticles at indicated time points. Data displays mean ± s.e.m. (*n* = 3). Statistical analysis was performed by two‐way ANOVA and Dunnett's multiple comparisons test. ****p < 0.0001. g) Graph exhibits percentage cytotoxicity of primed iBMDMs treated with blank nanoparticles at varying concentrations for 24 h, as measured by LDH Assay. Data shown here represents displays mean ± s.e.m. (*n* = 3). Statistical analysis was performed by one‐way ANOVA and Dunnett's multiple comparisons test. ****p < 0.0001.

**Figure** **2**a represents different components that self‐assemble to form FLTD‐DSR NPs. The inflammasome imaging ‐FLTD‐ probe and disulfiram drug, both being hydrophobic in nature, were physically encapsulated inside self‐assembling colipid nanoparticle backbone formed from DOPC and DSPE‐PEG (2000)‐Amine, by hydrophobic and hydrophilic interactions. The probe and drug specifically occupy the spaces between lipid bilayer while self‐assembling during hydration. The loading efficiency of FLTD probe in FLTD NP and FLTD‐DSR NPs ranged in between 70% and 80%, and that of disulfiram drug accounted for 60% in FLTD‐DSR NPs. The hydrodynamic diameter of FLTD and FLTD‐DSR NPs were evaluated using dynamic light scattering and were obtained to be 220 ± 10 and 160 ± 10 nm, respectively (Figure 2b and Figure S4a, Supporting Information). This data corroborated with the cryo‐transmission electron microscopy image of FLTD‐DSR NPs (Figure 2e) and size distribution histogram in Figure S5 (Supporting Information). After the size and morphology analysis, the nanoparticles were tested in PBS and serum for extended time points, to evaluate their stability in storage as well as physiological conditions. Their PBS stability was evaluated as a function of size and zeta potential, and both the particles were found to be stable in PBS for a period of 7 days as shown by their consistent attributes (Figure 2d; Figure S4b, Supporting Information). For in vivo applications, the particles were tested in physiologically relevant conditions, where they were incubated with 10% serum solution in PBS, at pH 7.4 and kept at 37 °C temperature for about 48 h, which correlates to circulating life of liposomal particles.^[^ ^33^ ^]^ FLTD‐DSR NPs exhibited serum stability for 48 h with no significant change in size and surface charge (Figure 2c). For inflammasome immunotherapy, it is critical to choose the appropriate nanoplatform which does not induce IL‐1*β* release by itself or after LPS priming of cells. In line with this, we strategically chose the colipids DOPC and DSPE‐PEG(2000)‐Amine as they have been previously proven to be biocompatible and have failed to stimulate IL‐1*β* in primed‐macrophages.^[^ ^21^, ^34^ ^]^ To reconfirm this, different concentrations of the colipid nanoparticles ((10–200) × 10^−6^ m) were incubated with primed macrophages for 12 and 24 h and their supernatant were assessed for IL‐1*β* levels using ELISA (Figure 2f). Moreover, these treated cells were also subjected to LDH assay so as to evaluate percent cytotoxicity and pyroptosis (Figure 2g). Both the graphs indicate that even at a higher concentration of 200 × 10^−6^ m, these particles do not induce IL‐1*β* release or cell death, making it a safer delivery tool for inflammasome immunotherapy. DSPE‐PEG (2000)‐Amine enhances the particle retention time by coating their surface with PEG which eventually prevents them from getting opsonized thus preventing subsequent clearance from the circulation.

**Figure 3:**
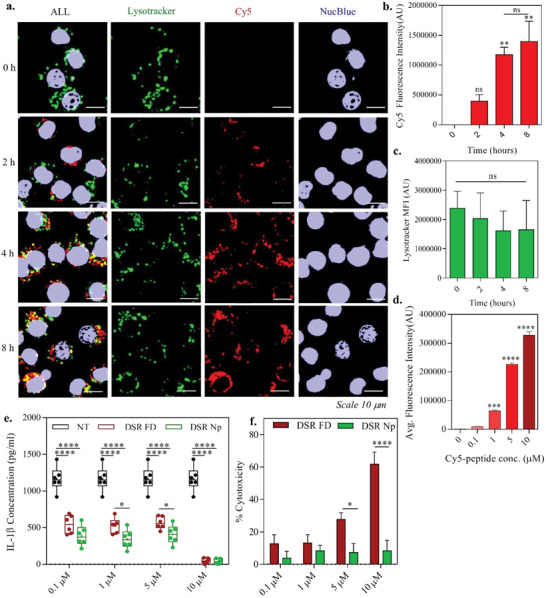
Internalization of fluorescent nanoparticles by iBMDMs. a) Representative confocal images of LPS primed iBMDMs treated with fluorescent nanoparticles encapsulated with Cy5‐tagged peptide (without QSY21) at indicated time points from 0 to 8 h. The nuclei were stained with NucBlue (shown in purple), and the lysosomes were stained with lysotracker Red DND 99. For better overlay representation, lysotracker is shown in green and Cy5 is shown in red. Colocalization is shown in yellow. Scale bar: 10 mm. b) Quantification of Cy5 fluorescence signal as a result of internalized nanoparticles over the period of 8 h at indicated time points. c) Graph plots the mean fluorescence intensity of lysotracker at the indicated time points. d) Internalization of Cy5 fluorescent nanoparticles at indicated concentrations in 4 h. Data in b, c and d are mean ± s.e.m. (*n* = 3). Statistical analysis was performed by one‐way ANOVA and Dunnett's multiple comparisons test. **p < 0.01, ***p < 0.001, ****p < 0.0001. e) Graph indicates IL‐1*β* levels in supernatant of nigericin treated cells pretreated with either disulfiram free drug or nanoparticle for 14 h. NT refers to no treatment with FD/NP. Data shown are min–max (*n* = 6). Statistical analysis was performed by two‐way ANOVA and Dunnett's multiple comparisons test. FD and Np were compared using Sidak's multiple comparisons test. *p < 0.05, ****p < 0.0001. f) Graph displays percentage cytotoxicity induced from disulfiram free drug or nanoparticle in primed iBMDMs after 14 h treatment. Data shown are mean ± s.e.m. (*n* = 6). Statistical analysis was performed by one‐way ANOVA and Dunnett's multiple comparisons test. *p < 0.05, ****p < 0.0001.

After synthesis and characterization of both the reporter FLTD and theranostic FLTD‐DSR nanoparticles, we next attempted to determine the internalization efficiency of these particles by macrophages. This was to identify optimum time point and the reporter dye concentration to obtain desired internalization so as to test the particle's efficiency in vitro in further assays. For all the in vitro experiments, we utilized immortalized bone‐marrow‐derived‐macrophages (iBMDMs) which have been extensively utilized to study inflammasomes in previous literature.^[^ ^20^, ^26^, ^35^, ^36^ ^]^ For internalization, iBMDMs were first primed with LPS for 2 h to mimic signal 1, and then stained with lysotracker so as to identify the mode of cargo delivery to the cytosol. Additionally, we were interested in determining if there is any lysosomal disruption that could potentially result in NLRP3 activation making it an unsafe platform to use as has been pointed out in previous studies.^[^ ^20^, ^37^, ^38^ ^]^ The particles utilized for internalization studies were engineered to be fluorescent by encapsulating just the Cyanine5‐peptide conjugate, without any quencher, which always keeps the signal “on.” As indicated in the representative images (**Figure** **3**a), LPS‐primed iBMDMs start to internalize the fluorescent nanoparticles from 2 h itself, showing significantly higher uptake at later time points (Figure 3b), which is similar to the observation from flow cytometry analysis (Figure S6, Supporting Information). Even though there is some colocalization of Cy5 and lysotracker at later time points indicating the uptake through endolysosomal pathway; there is no significant lysosomal disruption which could impact inflammasome signaling (Figure 3c). Lysotracker signal is relatively consistent in primed iBMDMs across the period of 8 h, indicating insufficient lysosomal disruption, which again proves this platform to be safe for inflammasome immunotherapy. As the nanoparticles do not induce lysosomal rupture, we were curious to discern the intracellular fate of FLTD NPs and FLTD‐DSR NPs by conducting dialysis release kinetics studies, which indicated that the liposome nanoparticles break down in late endosome/early lysosomal pH, which is extenuated in the biologically relevant macrophage lysate by a quicker burst release at the earlier time points. This pH responsive degradation would likely result in free probe/drug diffusion to the cytosol (Figure S7, Supporting Information). To identify the optimum concentration of the probe for further experiments, primed iBMDMs were incubated with DOPC nanoparticles delivering (0.1–10) × 10^−6^ m Cy5‐peptide in place of FLTD probe for 4 h, which accounts for a maximum of around 50 × 10^−6^ m of colipids. Figure 3d displays concentration dependent uptake of fluorescent NPs by LPS primed iBMDMs with 5 × 10^−6^ and 10 × 10^−6^ m exhibiting significantly higher fluorescence intensity. Together with the fact that colipids do not induce toxicity even at a higher concentration of up to 200 × 10^−6^ m, all the further experiments conducted with nanoparticles delivering providing 5 × 10^−6^ m reporter probe and nanoparticle concentrations were calculated keeping probe as the reference, so as to ensure its consistent delivery.

Next, we were interested in identifying what concentration range of disulfiram drug is safe to use in the theranostic FLTD‐DSR NP platform and is it as efficacious as that of free drug. In order to pursue that, we first treated the primed cells with disulfiram free drug or nanoparticle (single system) for 4 h, sufficient for internalization. Next, the nanoparticles were removed, and the cells were incubated in fresh LPS‐containing media for 10–12 h to continue priming and allowing sufficient time for the delivery of nanoparticle's drug to the cytosol and enable sustained response, with an aim to mimic physiological conditions. This was then followed by 10 × 10^−6^ m nigericin treatment for 1 hour as signal 2 to induce inflammasome assembly. After 1 hour of signal 2, supernatant was assessed for IL‐1*β* release and before nigericin treatment, cells were assessed for viability by MTT assay to examine the cytotoxicity of drug nanoparticle or free drug due to extended incubations. It was observed that as low as 100 × 10^−9^ m of DSR is sufficient to inhibit IL‐1*β* release by around 50% (Figure 3e) via both nanoparticle and free drug, with cells being around 90% viable before signal 2 treatment (Figure 3f). It was also observed that both the treatments display a comparable IL‐1*β* inhibition at different doses of DSR with 10 × 10^−6^ m showing the maximum response (Figure 3e). At 10 × 10^−6^ m DSR dose, we noticed a 100% IL‐1*β* inhibition via both nanoparticle and free drug, however, free drug also exhibited greater toxicity at higher doses (5× 10^−6^ and 10 × 10^−6^ m), reducing the cell viability to less than 50% in contrary to nanoparticle which maintained the viability to 90% throughout the concentration range of (0.1–10) × 10^−6^ m (Figure 3f), proving nanoparticle as most efficient due to sustained release effect. This also points to the fact that 100% inhibition shown by the free drug is partially deceptive because the effect is observed in only 40% of the viable cells after free drug treatment, whereas in nanoparticle the response is from 90% viable cells, which proves it as a better platform than just free drug. We confirmed that the delivery of around 5 × 10^−6^ m of the disulfiram using FLTD‐DSR NPs (corresponding to 5 × 10^−6^ m probe) will be sufficient to induce significantly greater response, which also accounts to be higher than the IC_50_ of 0.3 ± 0.01 × 10^−6^ m for NF*κ*B and GSDMD inhibition, thus will provide desired inflammasome inhibition.

**Figure 4:**
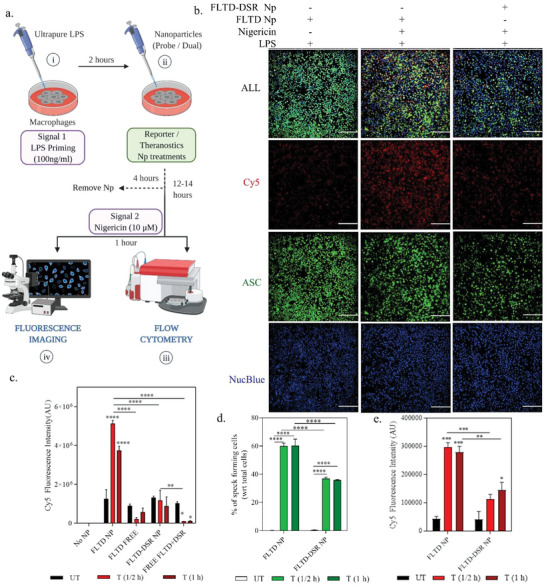
Nanoparticles enable efficient monitoring of inflammasome assembly and drug activity in nigericin treated iBMDMs. a) Schematics representation of reporter and theranostic activity test in vitro. iBMDMs were primed with 100 ng mL^–1^ of LPS as signal 1, which was followed by treatment with FLTD or FLTD‐DSR NPs for 4 h. After 4 h of NP treatments, nanoparticles were removed and fresh LPS containing medium were added. The cells were incubated overnight and further treated with 10 × 10^−3^ m nigericin as Signal 2, which was followed by either flow cytometry or fluorescence imaging at indicated time points. b) Representative confocal images of only primed and nigericin‐treated primed iBMDMs, pretreated with probe and probe‐drug nanoparticles. These cells contain citrine tagged ASC (green), and their nuclei were stained using NucBlue (blue). Cy5(red) indicates the signal from the caspase‐1 responsive probe (containing both Cy5 dye and QSY21 quencher). The images were captured after 1 hour of nigericin treatment. Scale bar: 200 mm. c) Quantification of Cy5 fluorescence intensity stimulated by treatments of different nanoparticles and free probes‐drugs combinations at indicated time points of 1/2 h and 1 h after nigericin treatment, via flow cytometry. d) Graph plots percentage of speck forming cells observed in confocal images of 1/2 h and 1 h of nigericin treatment in iBMDMs pretreated with nanoparticles. e) Microscopy quantitative analysis representing the average fluorescence intensity of reporter signal (Cy5) after 1/2 h and 1 h of nigericin treatments, exhibited by cells pretreated with probe and dual nanoparticles Data shown are mean ± s.e.m. (*n* = 3). Statistical analysis was performed by two‐way ANOVA and Dunnett's multiple comparisons test. **p < 0.01, ***p < 0.001, ****p < 0.0001.

For the activity studies, we utilized a similar approach as that of drug nanoparticle. LPS‐primed iBMDMs were treated with either FLTD or FLTD‐DSR NPs delivering either just 5 × 10^−6^ m reporter probe or an additional 5 × 10^−6^ m of disulfiram for 4 h. After 4 h of NP treatments, the cells were supplied with fresh media containing 50 ng mL^−1^ LPS to continue the priming (**Figure** **4**a). Next day 10 × 10^−6^ m nigericin was added after 12–14 h of overnight treatment, for signal 2 induction. Future experiments involved microscopy and flow cytometry for assessing the reporter activity, as well as western blotting to verify drug's activity. Fluorescence microscopy representative images display Cy5 reporter signal in nigericin treated cells pretreated with FLTD NPs which tend to reduce upon treatment with FLTD‐DSR NPs (Figure 4b) and this reporter signal was mostly concentrated around ASC specks or colocalized with them as shown in magnified fluorescence images (Figure S8, Supporting Information). The cyanine 5 signal is obtained from the reporter probe upon cleavage by active caspase‐1, which is verified by the utilization of *Casp1* ^−/‐^ iBMDM (Figure S9, Supporting Information). The caspase‐1 knockout iBMDMs exhibit almost negligible Cy5 signal upon pretreatment with reporter nanoparticles reassuring the reporter's specificity toward caspase‐1 activity (Figure S9a,b, Supporting Information). For microscopy, we utilized iBMDMs expressing citrine tagged ASC so as to corroborate the results obtained from reporter probe with the ASC speck formation, a hallmark for inflammasome assembly. The cells began to exhibit reporter signal at the first measured time point of 30 minutes after nigericin treatment as shown in the time‐dependent quantitative analysis of flow cytometry as well as microscopy images (Figure 4c,e). This signal was around 35% reduced when the cells were pretreated with FLTD‐DSR NPs due to drug's inflammasome inhibiting property leading to reduced caspase‐1 and gasdermin‐D activity, enabling the detection of drug activity early on. This response also corelated well with the ASC speck forming quantitation, plotted in Figure 4d. FLTD‐DSR NP pretreated cells exhibited around 50% lower ASC speck formation as compared to those treated with FLTD NPs, confirming that the drug was efficacious in inhibiting inflammasome assembly. To verify the drug's activity, we also examined the expression of different inflammasome‐associated proteins using western blotting (Figure S10a, Supporting Information). We observed a significant downregulation in expression of p20 caspase‐1 released into the supernatant (Figure S10a,b, Supporting Information), indicating the inhibition of procaspase‐1 cleavage to its active form, which concords with the microscopy observation of lower speck formation.

**Figure 5:**
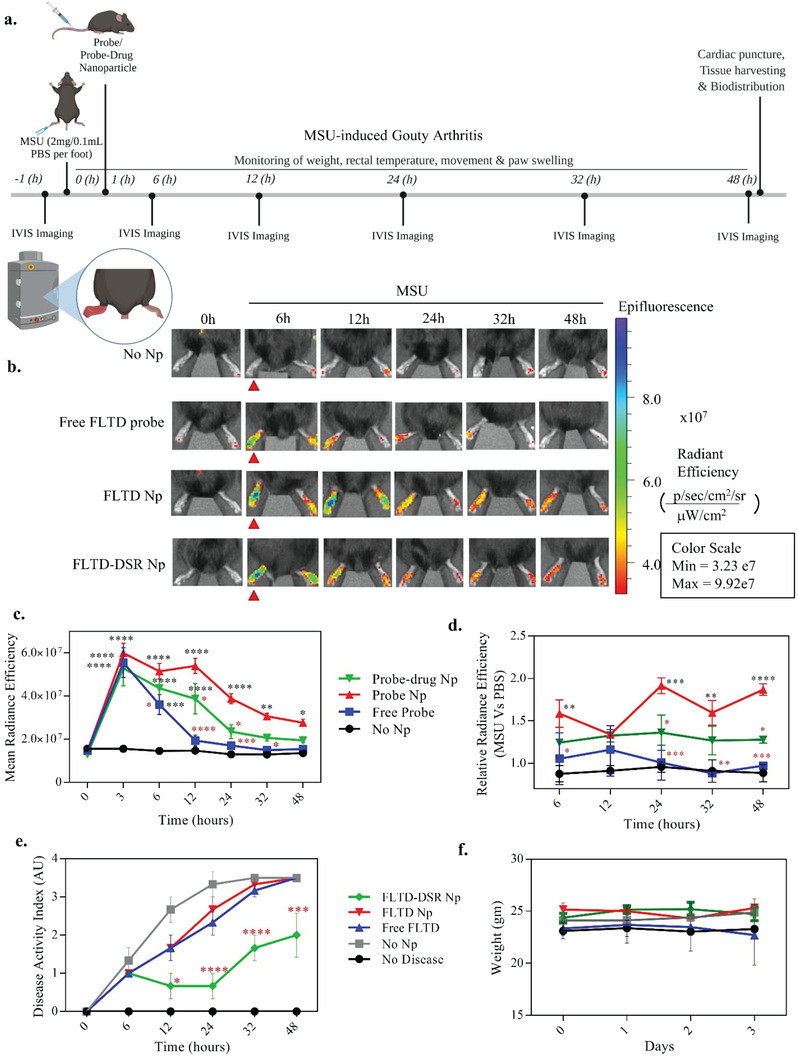
Reporter and theranostic nanoparticles efficiently track and treat MSU‐induced gouty arthritis via inflammasome targeted pathway. a) Schematic illustration demonstrating timeline for in vivo testing of reporter and theranostic nanoparticles in MSU‐induced gouty‐arthritis model. The timeline includes the time points for MSU footpad injection, nanoparticles or free probe‐drug intravenous injections, IVIS imaging, disease activity index monitoring and termination assays. b) Representative bright‐field and fluorescence overlap images of inflamed (MSU injected, red arrow) and uninflamed (PBS injected) foot at indicated time points after nanoparticle or free probe‐drug administrations, detected by in vivo imaging system (IVIS). The signal is from caspase‐1 cleavable reporter probe. c) The graph represents mean radiance efficiency of Cy5 fluorescence reporter signal from inflamed foot (left) of indicated treatment groups, exhibiting kinetics of nanoparticles and free probe monitoring efficacy for a period of 48 h. d) Graph plots relative radiance efficiency of fluorescence signal from inflamed versus normal foot across different treatment groups at indicated time points. e) Intensity of gouty arthritis is displayed as disease activity index for different treatment groups at indicated time points from 0 to 48 h. f) The graph displays time‐dependent weight measurements of mice from indicated treatment groups. Data in (c–f) are shown as mean ± s.e.m. (*n* = 5). Statistical analysis was performed by two‐way ANOVA and Dunnett's multiple comparisons test. **p < 0.01, ***p < 0.001, ****p < 0.0001. “*” indicates comparison from no disease group; “*” denotes the comparison from FLTD nanoparticle group.

Further, the reporter and theranostic nanoparticles were tested in MSU‐induced gouty‐arthritis model to assess their in vivo efficacy. Mice were subcutaneously injected with 2 mg monosodium urate dissolved in 100 µL PBS in left footpad to induce gouty arthritis, followed by FLTD or FLTD‐DSR NP injections intravenously (**Figure** **5**a).^[^ ^28^, ^39^, ^40^, ^41^ ^]^ This was followed by IVIS imaging at indicated time points from 0 to 48 h. It is evident from the representative in vivo imaging that the inflamed foot emits significantly higher signal in all the treatment groups when compared to PBS (Figure 5b). Upon comparing the signal kinetics in the inflamed foot over time among different treatment groups, it was observed that free FLTD probe showed a significant increase in signal at 3 h which immediately reduced in the subsequent hours (Figure 5c). Whereas in nanoparticle groups, the signal is sustained and remains for a subsequently longer time point as compared to the free probe. In FLTD NP treatment group, the signal was observed in a sustained manner until 12 h and relatively higher signal was observed at later time points as well. In comparison to this, FLTD‐DSR NP treatment group showed a significantly lower signal at 12 and 24 h, showing the efficacy to track the treatment early on. Although there is some signal in the uninflamed foot which might be because of the basal level of signal or signal due to localized inflammation from wounds or scratches, there is still a relatively higher signal in MSU injected foot as displayed in the Figure 5d. In the relative radiance efficiency plot, FLTD NP showed a significantly higher signal as compared to all other groups at indicated time points. This signal was relatively reduced upon utilizing the FLTD‐DSR NP as a result of drug's activity which was verified using the disease activity index plot. The gout scoring included several criteria like movement, food or water intake, posture, and paw swelling, which was taken into consideration for plotting the graph for disease activity index (Figure 5e). Here we observed in FLTD‐DSR NP group the treatment started to work in 12 h itself which corroborated with the decreased signal observed from the reporter. None of the treatment groups were toxic or had any adverse effects as can be seen in their weights (Figure 5f) as well as hematoxylin and eosin (H&E) stains of vital organs showing normal pathologies in the heart, liver, spleen, lung, and kidney tissues (Figure S11, Supporting Information). The nanoparticle systems were also shown to be highly bioavailable in the relevant foot tissue over 24 h, as indicated by replacing FLTD in the FLTD NP with a noncleavable Cy5 reporter for biodistribution (Figures S12 and S13, Supporting Information). We also sought to determine the PK/BD of the release of DSR by only drug DSR NPs. To do this, we injected 10 mg kg^−1^ doses of DSR‐NPs in C57BL/6 mice, and sacked them at 0, 2, 6, 12, 24, and 48 h timepoints, collecting blood by heart puncture and liver, lung, kidney, spleen, and heart tissues to determine the concentration of DSR by reverse phase liquid chromatography in a C18 column followed by mass spectrometry analysis. From the LCMS results, shown in Figure S14 (Supporting Information), we discern an interesting pattern of biodistribution and clearance of DSR from the nanoparticles. Most notably, after tail vein injection of the NPs, the initial serum concentration of roughly 125 ng mL^−1^ is cleared quickly. After 2 h, the concentration of serum decreases by more than half, and is reduced to below the level of the blank at the 6 and 12 h timepoints, besides a resurgence at 24 and 48 h which is presumed to be an issue of background signal or retention time in the column. However, interestingly, the DSR in the serum is not immediately cleared by the liver. Instead, the highest concentration of DSR is found in the kidney and spleen during the early time points, most notably at 2 h. After 12 h we observed the highest concentration of DSR in the liver, and surprisingly the lung, the former indicating that the DSR NPs have begun to be taken up by the liver and the DSR cleared from the body.

**Figure 6:**
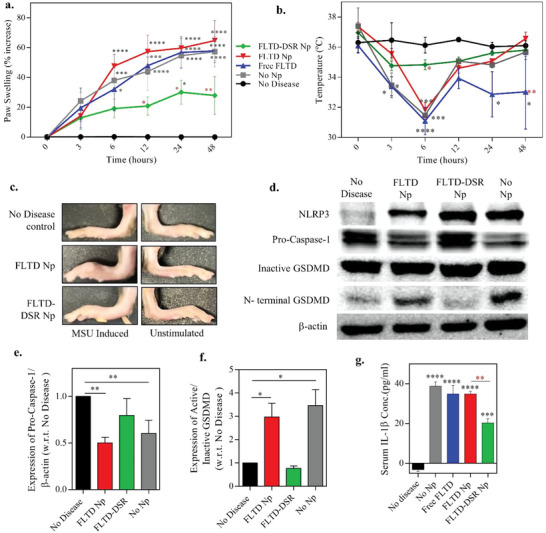
Studies displaying the efficacy of inflammasome inhibiting drug treatment stimulated by theranostic nanoparticles. a) Graph represents percentage increase in paw swelling of inflamed foot as compared to normal foot upon indicated treatments of nanoparticles or free probe for a time period of 48 h. b) Rectal temperature of mice at indicated time points measured during 48 h after administration with specified treatments. Data in a and b are shown as mean ± s.e.m. (*n* = 5). Statistical analysis was performed by two‐way ANOVA and Dunnett's multiple comparisons test. **p < 0.01, ***p < 0.001, ****p < 0.0001. “*” indicates comparison from no disease group; “*” denotes the comparison from FLTD nanoparticle group. c) Representative swelled paw of MSU stimulated (left) and unstimulated (right) foot of mice treated with reporter and theranostic nanoparticles. d) Western blot representative of different inflammasome‐associated protein expression‐ NLRP3, procaspase‐1 and GSDMD in homogenized lysate of paw tissue obtained from mice treated with indicated nanoparticle or free probe. b‐actin was used as the house‐keeping control. e) Relative expression of active caspase‐1 versus procaspase‐1 quantified from western blotting analysis of paw tissue extract from mice treated with indicated nanoparticles or free probe. f) Quantification of relative expression of active GSDMD versus inactive GSDMD in paw tissue lysate of mice from specified treatments after sacrifice. g) Concentration of IL‐1b cytokine in serum isolated from mice belonging to specified treatment groups upon sacrifice. Data in (e–g) are shown as mean ± s.e.m. (*n* = 3). Statistical analysis was performed by one‐way ANOVA and Dunnett's multiple comparisons test. **p < 0.01, ***p < 0.001, ****p < 0.0001.

For additional measures of therapeutic efficacy, we also measured the paw swelling using vernier caliper, by quantifying percentage increase in inflamed foot size as compared to uninflamed one in three regions, toes, ankle, and middle paw area. **Figure** **6**a shows that the swelling was reduced by a third upon using the FLTD‐DSR NP exhibiting its efficacy over time. The significant decrease in swelling upon drug treatment could also be seen in the representative images obtained at the end of the trial (Figure 6c). These results also aligned with the dip observed in the body temperature of mice from different treatment groups measured over time and displayed in Figure 6b. Next, we identified the expression of different inflammasome‐associated proteins in the inflamed foot extracts across different groups using western blotting to reconfirm the treatment efficacy. As shown in Figure 6d, there is significantly higher NLRP3 expression in all the treatment groups as compared to the no disease control group. However, procaspase‐1 is significantly inhibited in FLTD NP and no nanoparticle groups, pointing toward a higher cleavage of inactive caspase‐1 to its subsequent active form. Whereas there is higher expression of procaspase‐1 observed in no disease and FLTD‐DSR NP groups denoting lesser cleavage activity and thus, lower active caspase‐1 expression (Figure 6d,e). The N‐terminal GSDMD, denoting the active form, around 37 kDa, is strongly inhibited upon treating the infected mice with FLTD‐DSR NPs which is similar to what we observed in no disease control, confirming again that the treatment worked at the termination of the animal trial (Figure 6d,f). Similar results were also observed in the serum IL‐1*β* levels, where probe‐drug showed around 50% lower response as compared to other treated groups (Figure 6g). Finally, we were also able to confirm therapeutic efficacy of FLTD‐DSR NPs through hematoxylin & eosin (H&E) staining done on sections from the control foot of PBS injected mice and gouty arthritic foot of mice treated with FLTD only NP and FLTD‐DSR NP (Figure S15, Supporting Information). From these images, we have discerned that the intense inflammation in gouty arthritic joints of FLTD NP treated mice, were categorized by extensive necrotizing inflammatory infiltration composed of neutrophils, lymphocytes, and plasma cells, with histocytes in the periphery of the necrotized area. This was greatly ameliorated upon treatment with FLTD‐DSR NPs, due to inflammasome inhibiting response of DSR.

## 3 Conclusion

In summary, we have demonstrated the synthesis of reporter and theranostic nanoparticles facilitated by the self‐assembly of colipids. The nanoparticles were stable in both storage and physiological conditions for extended time periods as indicated by minimal or no change in their average size and zeta potential. Reporter nanoparticles (FLTD NPs) were successfully able to exhibit spatiotemporal activation of inflammasome complex in in vitro conditions. Additionally, theranostic nanoparticles (FLTD‐DSR NPs) showed robust drug's activity in vitro which enabled early detection of treatment efficacy via reporter probe. These results were further validated in an MSU‐induced gouty‐arthritis model, where enhanced reporter NIR signal was observed in the group treated with FLTD NPs and the signal was sustained until later time points as compared to the free probe treatment signal which instantly peaked at 3 h and reduced afterward. In comparison, FLTD‐DSR NPs showed relatively reduced signal due to efficient drug's activity, which was also validated from disease activity index, paw‐swelling measurements, rectal temperature, serum IL‐1*β* levels, expression of procaspase‐1 and active N‐terminal GSDMD. Mice body weight measurements and H&E of vital organs showed no significant changes across different treatments groups pointing toward the fact that the doses were nontoxic. Furthermore, the liposomal formulations exhibited sufficient pharmacokinetics and biodistribution of both probe and drug in the physiologically relevant tissues, supporting our findings. Overall, both the platforms together facilitated real‐time monitoring of inflammasome complex assembly and its targeted therapy in vitro as well as in vivo. This diagnostic and theranostic platform pair efficiently enable spatiotemporal imaging of inflammasome activation and predicts treatment efficacy of inflammasome targeting via longitudinal imaging.

## Conflict of Interest

The authors declare no conflict of interest.

## Supporting information

Supporting Information

## Data Availability

The data that support the findings of this study are available from the corresponding author upon reasonable request.
