# Supplementary material for: Nanoreporter for Real‐Time Monitoring of Inflammasome Activity and Targeted Therapy
Source: Adv Sci (Weinh). 2023 Jan 5;10(6):2204900. doi: 10.1002/advs.202204900 (PMC9951342; doi:10.1002/advs.202204900)
Supplement: Supplementary file 1 — Supporting Information [file ADVS-10-2204900-s001.pdf]

Supporting Information

Nano-reporter for Real-time Monitoring of Inflammasome Activity and Targeted Therapy

Dipika Nandi[1,2], James Forster[1], Anujan Ramesh [1, 3], Anh Nguyen[1], Hariharan Bharadwaj[5], Ashish Kulkarni[1,2,3,4]*

[1]Department of Chemical Engineering, University of Massachusetts, Amherst, Massachusetts 01003, USA.

[2]Department of Veterinary and Animal Sciences, University of Massachusetts, Amherst, Massachusetts 01003, USA.

[3]Department of Biomedical Engineering, University of Massachusetts, Amherst, Massachusetts, 01003, USA

[4]Center for Bioactive Delivery, Institute for Applied Life Sciences, University of Massachusetts, Amherst, Massachusetts 01003, USA.

[5]Department of Pathology, UMass Chan, Medical School-Baystate, Springfield, MA 01107, USA

*Correspondence to: akulkarni@engin.umass.edu

Materials and Methods

Materials

All the reagents were procured from commercial suppliers including ThermoFisher, InvivoGen, Tocris, Avanti, Adipogen, Biolegend, BioRad, Cell Signaling Technology, Biorad and Sigma-Aldrich. FRET pair probe Cy5-KFLTDK(QSY21)-C were custom ordered from CPC Scientific with 95% purity (Cat# 940007). Nanoparticle components 1,2-Dioleoyl-sn-glycero-3-phosphocholine (DOPC, Cat# 850375P) and 3-phosphoethanolamine-N-[amine (polyethylene glycol)-2000] (DSPE-PEG2000Amine, Cat# 880128P) were purchased from Avanti Polar Lipids. Disulfiram drug, also known as Bis(diethylthiocarbamyl) disulfide (Cat# 97-77-8), was obtained from Millipore Sigma. For cell-based assays, all the immortalized bone-marrow-derived-macrophages (iBMDMs) including the cell lines expressing ASC-citrine and knockout for Caspase-1 were generously gifted by Kate Fitzgerald, UMass Chan Medical School, Worcester. Cell culture media components such as Dulbecco's modified Eagle's medium (DMEM), heat-inactivated fetal bovine serum (FBS), and Penicillin-Step were ordered from Gibco, Life Technologies. For inflammasome stimulation, ultrapure lipopolysaccharide from *Escherichia coli* (Ultrapure LPS, E.coli 0111:B4, Cat# tlrl-3pelps) and Nigericin sodium salt (Cat# 431210) were obtained from InvivoGen and Tocris Bioscience, respectively. All the western primary and secondary antibodies were procured from Adipogen Life Sciences, Biolegend, Abcam, Santa Cruz Biotechnology and Cell Signaling Technology. Other reagents like RIPA buffer, NP-40 lysis buffer, HALT protease and phosphatase single-use EDTA-free 100X cocktail, J60015 4X Laemmli SDS reducing sample buffer, NucBlue Live Ready Probes Reagent (Hoechst 33342), and LysoTracker Red DND-99 were purchased from ThermoFisher Scientific. Moreover, some kits

like IL-1 beta Mouse Uncoated ELISA and Pierce BCA Protein Assay were also obtained from ThermoFisher Scientific. TGX Stain-Free FastCast Acrylamide Kit, 10% and Clarity Western ECL substrate were ordered from Biorad. For animal studies, Monosodium Urate Crystals (MSU, Cat# tlrl-msu-25) were purchased from InvivoGen. All the needles, syringes, and serum separator BD Microtainer capillary blood collector (Cat# 02-675-185) were purchased from Fisher scientific. All other chemicals and reagents were purchased from either Sigma-Aldrich or Fisher Scientific. Glass wares and plastic wares were also ordered from Fisher Scientific. For LCMS experiments, Kinetex® XB C_{18} column was procured from Phenomenex (Torrance, CA, USA), and solid phase extractor SOLA SPE reverse phase cartridges were procured from Thermo Fisher.

Detailed Methods

Synthesis of Nanoparticles FLTD Np, FLTD-DSR Np and DSR Np. The supramolecular nanoparticles, reporter and theranostic NPs, were synthesized using the lipid-film hydration method. 5% of -FLTD- imaging probe, 5% of disulfiram, 30 mol % of 1,2- distearoyl-sn-glycero-3-phosphoethanolamine-N-[amine (polyethylene glycol)-2000] [1] and 60 mol % of 1,2-Dioleoyl-sn-glycero-3-phosphocholine were dissolved in 1ml of DCM. For single reporter only or drug only nanoparticles, 10 mol% of either -FLTD- imaging probe or disulfiram was used. The rotary evaporator was used to evaporate the solvent, so as to form a thin and uniform film. This thin film was further hydrated with ultrapure PBS for 1.5 hours at 60°C to obtain self -assembled supramolecular nanoparticles. Next, the particles were eluted through a G-25 sephadex (Cat# G25150) column, which was prepared using swelled sephadex prepared by heating them in MilliQ for 1 hour. The filtered nanoparticles were then sequentially extruded through a 0.4 μm and 0.2 μm polycarbonate membrane to obtain a size of less than or around 200 nm. The hydrodynamic

diameter was measured using Dynamic Light Scattering (DLS) and the encapsulating efficiency was determined by UV-Vis spectroscopy. The drug loading efficacy was calculated as a percentage of drug recovered from nanoparticle fractions in reference to the initial amount taken.

Stability studies of reporter and theranostic nanoparticles in PBS and human serum. To determine the physical stability of the FLTD NP and FLTD DSR NPs at storage conditions, they were incubated in PBS at 4°C and tested for average particle size and zeta potential every day, by Dynamic Light Scattering method, upto a time period of 7 days. The physiological stability of these particles was determined by incubating freshly synthesized FLTD-DSR NP in 10% human serum for 48 hours, continuously stirred at 37°C. The serum stability was plotted as a function of percentage change in particle size and zeta potential at indicated time points, measured by Dynamic Light Scattering method using Zetasizer Nano ZS90 (Malvern, UK).

Cryo-TEM. Samples were prepared using a Vitrobot and liquid ethane supported onto plasma-treated lacey carbon 400-mesh copper grids. 5 µL of nanoparticle suspension diluted in PBS at a ratio of 1:1000 was applied onto the plasma-treated grids. They were further blotted with filter paper and processed by vitrification in liquid ethane. Transmission electron microscopy was performed using a Phillips CM 120 Cryo operating at 120 keV using a Gatan Oris 2k by 2k CCD camera system. To maintain the grids at a very low temperature (below -180°C), they were transferred into the cryo-electron microscope using a cryostage. Images were obtained at 57 kx under low-dose conditions at 8-10 electrons Å2. TEM images were then measured for particle size using ImageJ.

Probe testing and validation using caspase-1 enzyme. Recombinant mouse Caspase-1 protein (abcam, ab52079) was first reconstituted in PBS containing 15% glycerol to obtain a stock solution of 1U/µL, which was stored in -80°C for long-term. Then, a highly concentrated reporter stock solution, 500 µM, was prepared by dissolving -FLTD- imaging probe in DMSO, which was further diluted to 100 µM using caspase assay buffer (50 mM Hepes, pH 7.2, 50 mM NaCl, 0.1% Chaps, 10 mM EDTA, 5% Glycerol, and 10 mM DTT). To test the reporter activity, first 40 µL of caspase assay buffer was pipetted into 96 well black clear bottom microtiter plate and kept at room temperature to allow equilibrium to assay temperature. This was followed by addition of 10 µL/10U caspase-1 (1U/µL) to each treatment well and PBS to control wells. Finally, 50 µL of 100 µM -FLTD- reporter probe was added to all the wells so as to obtain its final concentration of 50 µM and allowed to incubate over the course of 24 hours at 37°C. For determining the specificity of probe, exact same conditions were applied to test recombinant caspase-3 enzyme activity, where 50 µM of -FLTD- probe was incubated with 10U of active caspase-3. Fluorescence intensities for both caspase-1 and caspase-3 were recorded every 15 minutes until first 2 hours, followed by every 30 minutes until next 22 hours, using a BioTek plate reader at Cyanine-5 excitation/emission.

Cell culture, inflammasome stimulation and nanoparticle treatments. Immortalized bone-marrow-derived macrophages (iBMDMs), iBMDMs expression ASC-Citrine, iBMDMs deficient in caspase-1 (*Casp1*$^{-/-}$ iBMDMs) were graciously gifted by Dr. Kate Fitzgerald from UMass Chan Medical School, Worcester. Immortalized cell lines were obtained from C57BL/6 primary BMDMs by utilizing J2 transforming retroviruses.[2],[3] The cells were cultured in DMEM supplemented with 10% heat-inactivated fetal bovine serum (FBS) and 50 µg/mL each of penicillin as well as streptomycin. They were passaged using 0.25% trypsin diluted in PBS. For all the in-vitro assays, iBMDMs were first stimulated with 50 ng/mL ultrapure lipopolysaccharide (LPS)

(InvivoGen, Cat# tlrl-3pelps) for 2 hours to prime them for signal 1. This was followed by treatment with different nanoparticles at indicated concentrations for 4 hours in LPS containing complete DMEM media so as to allow their cellular uptake. Then the nanoparticles were removed, and the cells were incubated in fresh LPS containing media for another 10-12 hours to continue priming and allow the sustained release of probe or drug. For signal 2, primed cells were treated with 10 μM nigericin (Tocris Bioscience, Cat# 43-1210) for indicated time period ranging between 30 minutes to 2 hours before any specific assays. Different signal 2 time points were utilized for different experimental protocols.

Cellular uptake of Cy5-peptide encapsulated NPs. To assess the cellular uptake of nanoparticles, iBMDMs were first primed with 50 ng/mL LPS for 2 hours and then treated with fluorescent nanoparticles in LPS containing media for different time points ranging from 0 to 8 hours or varying concentrations from 0.1 to 10 μM. The fluorescent nanoparticles utilized in this study were synthesized by physically encapsulating Cyanine5-tagged-peptide without any quencher to mimic the reporter probe nanoparticle that doesn't require activation and allow continuous fluorescence due to lack of quencher.

For microscopy, 0.8 million cells were seeded in an 8 well-chamber plate, after adherence they were treated with above stimulations. This was followed by incubation with 1 μM lysotracker DND-99 (ThermoFisher, Cat# L7528) and 2 drops of NucBlue (ThermoFisher, Cat# R37605) per mL of media for about 20 minutes, and imaging using Crest V2 Spinning disk. Lysotracker staining was performed with an aim to determine the nanoparticle localization within lysosomes and to identify the degree of lysosomal disruption following it, which were quantified using NIS Elements AR (Ver.4.50).

For flow cytometry, 1 million cells were seeded in a 12-well plate and primed with LPS for 2 hours. After LPS priming the cultured cells were treated with 5 μM fluorescent nanoparticles at indicated

time points and washed with 1X PBS twice. After washings, the stained cells were resuspended in 100 μL of FACS-staining buffer (1X PBS, 10% FBS, 01% NaN3 sodium azide) and quantified using ACEA Novocyte flow cytometer. Finally, Cy5 positive cells were analyzed and quantified using NovoExpress 1.2.5 software. For concentration dependent studies, the cells were treated with varying concentrations of fluorescent nanoparticles, 0.1 to 10 μM. This was followed by 2 times PBS washings, processing in FACS staining buffer and data acquisition as well as quantification using ACEA novocyte flow cytometer.

Dialysis of Nanoparticles to Determine Release Kinetics and Intracellular Fate

To determine the route at which the nanoparticles induced sufficient probe and drug release, we conducted a dialysis release kinetics experiment using DOPC FLTD nanoparticles synthesized as previously but substituting FLTD probe with 5 mol% DiD dye, a lipohilic carbocyanine dye. To test DiD release over time, indicative of nanoparticle breakdown, 500 μL of DiD encapsulating NPs were diluted in 1.5 mL of PBS at pH 7.4, 5.5, and 4.5, and dialyzed against a bath of the same pH in Spectrum™ Spectra/Por™ Float-A-Lyzer G2 Dialysis Devices (3.5-5k MWCO) for 8 hours. At time intervals of 0h, .5h, 1h, 2h, 4, and 8h, 100μL of sample was taken from the dialysis bag and absorbance was measured at 663 nm using a BioTek plate reader. Cumulative release was determined by subtracting the absorbance of each timepoint by the blank, and normalizing based on the initial 0h absorbance. This protocol was also repeated by diluting the nanoparticles in macrophage lysate and dialyzing against PBS at pH 4.5 and 5.5, to simulate the effect of late endosome/lysosome enzymatic environments on the nanoparticle system.

Cell-Viability Assays- LDH Assay & MTS Assay. We performed either LDH assay or MTS assay to determine the cell death of the iBMDMs treated with nanoparticles. LDH Assay was used to calculate the percentage cytotoxicity of blank nanoparticles (co-lipid nanoparticle). For this assay, cells were plated at a density of 8×10^4 cells per well in a 96-well plate and were primed with 50 ng/mL LPS for 2 hours. This was followed by treatment with different concentration of nanoparticles, ranging from 10 to 200 μM of co-lipids, for 4 hours. After the nanoparticle treatment, the cells were replaced with fresh LPS-containing basal media and positive control group was treated with 10 μM of nigericin, 1 hour before the supernatant collection. The collected supernatant was analyzed for percentage LDH release using invitrogen CyQUANT LDH Cytotoxicity Assay Kit (Cat# C20300), according to the manufacturer's instructions. Percentage cytotoxicity was calculated using the formula: % Cytotoxicity or % LDH release = {(Nanoparticle-treated LDH activity) - (Spontaneous LDH activity)}/{(Maximum LDH activity obtained via Triton X-100) - (Spontaneous LDH activity)} × 100.

To determine the cell cytotoxicity after treatment with drug nanoparticle (DSR Np) or free drug (DSR FD), Promega CellTiter 96 Aqueous One Solution Cell Proliferation Assay (MTS) (Cat# G3582) was used. To measure the viability, the nanoparticle-treated cells were first washed with PBS and replaced with colorless basal media followed by incubation in 1:20 ratio of MTS [3-(4,5-dimethylthiazol-2-yl)-5-(3-carboxymethoxyphenyl)-2-(4-sulfophenyl)-2H-tetrazolium] and PMS (Phenazine methosulfate) solution for 30 minutes. Absorbance was observed at 490 nm O.D. using BioTek plate reader.

Measurement of cytokine release using ELISA. For *in-vitro* analysis of cytokine release, supernatant from the treated iBMDMs were collected and subjected to IL-1β ELISA (ThermoFisher, Cat# 88-7013-76) according to the manufacturer's protocol.

For *in-vivo* experiments, the blood was collected from mice using cardiac puncture. The collected blood was immediately transferred to serum separation tubes (BD microtainer, Cat# 02-675-185) and allowed to clot for 30 minutes. Then the tubes were centrifuged at 6000-15,000 g for 1-3 minutes to separate the serum. The separated serum was collected in a fresh eppendorf tube and either stored at -80°C or directly used for ELISAs to quantify IL-1β cytokine levels. All the ELISAs were performed using the manufacturer's protocol.

Confocal Microscopy to monitor in-vitro efficacy of NPs. For in-vitro activity analysis of reporter/theranostic nanoparticles, two types of cell-lines were used, iBMDMs expressing ASC tagged with citrine and iBMDMs deficient in Caspase-1. For examining the *in-vitro* efficacy, 0.5 $\times 10^5$ iBMDMs expressing ASC-citrine were seeded per well of an eight-well chamber slide (Ibidi, Cat# 80806). Whereas to verify the caspase-1 specificity of the reporter nanoparticles, *Casp1*$^{-/-}$ iBMDMs were used. After adherence the cells were primed with LPS, treated with indicated nanoparticles for 4 hours, followed by incubation in fresh LPS-containing media for 12 hours and 10 µM nigericin treatment. 2 drops of NucBlue per ml of media was added along with nigericin to stain the nuclei. The images were taken at ½ and 1 hour of nigericin treatment using Nikon CrestV2 spinning disk and analyzed using NIS Elements 4.6.

Flow Cytometry to monitor in-vitro efficacy of NPs. To examine the *in-vitro* efficacy of the NPs using flow cytometry, 0.5-1 $\times 10^6$ iBMDMs were plated per well of a 12-well plate. They were

subjected to similar treatments for signal 1, nanoparticles and signal 2 as described earlier. After the respective treatments at indicated time points, the cells were washed with 1X PBS twice. Next, they were scrapped and resuspended in 100 µL of FACS staining buffer, which was followed by sample acquisition and quantification using ACEA Novocyte flow cytometer. The data was later analyzed using NovoExpress 1.2.5 software.

Animals. All animal experiments were approved by and performed in compliance with the guidelines of Institutional Animal Care Use Committees (IACUC) at the University of Massachusetts, Amherst. Male C57BL/6 mice of age 10-12 weeks were procured from Charles River and housed at 24 ± 2 ºC under 12 hours light or 12 hours dark cycles in a specific pathogen free (SPF) environment with free access to food and water.

MSU-Induced gouty arthritis model. Monosodium urate procured from InvivoGen (Cat# tlrl-msu-25) was dissolved in PBS at the concentration of 20 mg/mL and sonicated for 15 minutes to obtain a homogenous milky solution. To induce gouty arthritis, left hind footpad of the mice (dorsal up) was subcutaneously injected with 2 mg monosodium crystals prepared in 100 µL of PBS. Immediately after MSU injections, they were intravenously injected with the reporter or theranostic nanoparticles or free probe and imaged at 3, 6, 12, 24, 32 and 48 hours. They were also closely monitored for weight, temperature, body posture, movement and paw swelling at indicated time points.

Disease Activity Index. Hind paw thickness was measured using a vernier caliper and arthritis visual scores were established on the basis of tarsal/ankle oedema and erythema presence (0 = no

arthritis, 1 = slight swelling and/or erythema, 2 = moderate swelling/erythema, 3 = severe oedema/erythema and 4 = excessive oedema spanning all over the paw). Mice were also closely monitored for body temperature, weight, posture, and movement.

IVIS Imaging to monitor in-vivo efficacy of NPs.

After inducing the mice with gouty arthritis and injecting them with FLTD/FLTD-DSR NPs as explained earlier, they were imaged using PerkinElmer IVIS Spectrum system at 3, 6, 12, 24, 32 and 48 hours. The stage settings for animal imaging were kept consistent across the entire study. The emission filter range was set from 600-640 nm and the excitation range was 670-720nm. At the end of the study the excised organs including liver, kidney, spleen, lung and heart were imaged using the same settings as those of live mice. The NiR images were analyzed using PerkinElmer Live Image Software using spectral unmixing to eliminate the autofluorescence at the site of inflammation as well as from the organs. For spectral unmixing, a library was created using guided method, which was applied to all the raw images of live mice as well as organs imaging. Same radiance scale was maintained for the whole analysis, and each radiance signal was normalized from the tissue background signal. The graphs were plotted displaying the average radiance efficiency of each treatment group in a time-dependent manner, as well as for relative radiance efficiency of MSU injected hind foot compared to the uninflamed hind foot.

Biodistribution of Cy5-peptide fluorescent nanoparticles. C57BL/6 mice were injected with Cy5-peptide encapsulated fluorescent nanoparticles so as to determine its biodistribution in inflamed foot over time and identify its distribution in other organs such as liver, spleen, kidney, lungs and

heart after 24 hours. All the live mice and organ imaging was done using PerkinElmer IVIS Spectrum system at a filter range set at Cy5 excitation and emission range.

In vivo Pharmacokinetic Analysis of DSR from Nanoparticles via Liquid Chromatography – Mass Spectrometry (LCMS)

To address the pharmacokinetics and biodistribution of the nanoparticle system with respect to the release of disulfiram (DSR) drug in vivo, we tail vein injected C57BL/6 mice with DSR-NPs (FLTD-DSR NPs replacing FLTD probe with additional DOPC lipid) at a DSR dosage of 10 mg/kg. At the time points 0h, 2h, 6h, 12h, 24h and 48h, two mice were sacked, blood was isolated form mice through cardiac puncture, and the liver, lungs, kidney, heart and spleen were isolated and stored in -80ºC. Serum was separated from blood using serum-separation tubes, while 50 mg of the organs was carefully weighed out and pulverized using a homogenizer in 200 µl of RIPA buffer. The debris were then pelleted, and the supernatant was isolated and subsequently solid phase extraction was performed. SPE was performed in Thermo Fisher SOLA SPE reverse phase cartridges. The cartridge was conditioned with 1 mL methanol followed by equilibration with 1 mL 0.05 M EDTA. Then, either 50 µL of serum or 200 µl of the supernatant from homogenized organs were loaded into the cartridge and was washed with 500 µL water (containing 5% acetonitrile) followed by elution with 500 µL acetonitrile. Then, 500 µL eluent was collected in a clean glass tube for chromatographic separation and mass spectrometry analysis.

Chromatographic separations were carried out on a Phenomenex Kinetex® XB C_{18} column (50 mm × 21 mm, 2.6 µm; Phenomenex, Torrance, CA, USA) with a run time of 3 min. The temperature of the column and auotosampler was held constant at 40 °C and 4 °C, respectively. The mobile phase used for gradient elution consisted of (A) acetonitrile and (B) water (containing 0.1% formic acid and 1 mM ammonium acetate) at a flow rate of 0.2 mL/min. The gradient conditions of

the mobile phase were as follows: (A) was increased linearly from 20% to 80% during the first 0.8 min and held at that value for 1.4 min, then returned to the original ratio in the same way and re-equilibrated for 0.5 min. The injection volume was 50 μL. The mass spectrometer was operated in positive ion mode.

Immunoblotting. To prepare the western lysate, iBMDMs were plated at a density of 8×10^6 in 10 cm^2 plates. Adhered cells were then primed with LPS for 2 hours, followed by nanoparticle treatments for 4 hours. The cells were replaced with fresh LPS-containing media for 10-12 hours and later treated with 10 μM nigericin for 2 hours. After signal 2 treatment, the cells were washed with PBS twice and lysed using RIPA lysis buffer (ThermoFisher, Cat# 89900) enriched with 1X protease and phosphatase HALT inhibitor (ThermoFisher, Cat# 78440). Cell lysates were then subjected to high-speed centrifugation, 14,000 rpm for 15 minutes at 4°C, and the total protein extract collected in the supernatant was transferred to a fresh tube and stored in -80°C for long term storage.

For the tissue lysate preparation, 5 mg of excised foot was homogenized and lysed using 500 μL NP-40 lysis buffer (ThermoFisher, Cat# J60766-AP) enriched with 1X protease and phosphatase HALT inhibitor. Lysate was further centrifuged at 10,000 rpm for 10 minutes at 4°C to obtain the protein extract in supernatant. The isolated protein concentration is estimated using Pierce BCA (Bicinchoninic acid assay) Protein Assay kit by ThermoFisher Scientific (Cat# 23225). For immunoblotting, equal amount of estimated protein were separated by sodium dodecyl sulfate polyacrylamide gel electrophoresis (SDS-PAGE) on a 10−12% polyacrylamide gel and then transferred to a poly(vinylidene fluoride) (PVDF) membrane. The transferred blot was blocked with 5% skim milk in TBST, followed by incubation in primary antibodies of anti- caspase-1 (1:1000), anti-NLRP3 (1:1000), and anti-β-actin (1:2000) in 1% BSA TBST solution for overnight at 4 °C. Next, the

membranes were incubated with HRP-conjugated secondary antibody (1:2000) at room temperature for an hour. Primary antibodies anti-Caspase-1 p20 mouse mAb (Cat# AG-20B-0042-C100) and anti-NLRP3/NALP3 mouse mAb (Cat# AG-20B-0006-C100) were purchased from Adipogen Lifesciences. Anti-GSDMD antibody (Cat# ab209845) was procured from Abcam. Anti-β-actin antibody (Cat# 643802), anti-mouse IgG HRP-conjugated antibody (Cat# sc-516102) and anti-rabbit IgG HRP-linked antibody (Cat# 7074) were obtained from Biolegend, Santa Cruz Biotechnology and Cell Signaling Technology, respectively. Biorad ECL Clarity (Cat# 1705061) was used to detect the immunoreactive bands by Biorad ChemiDoc Imaging Systems. For image analysis and quantification of densitometric values, ImageJ software was employed.

Hematoxylin & Eosin (H&E) Staining for Efficacy and Biosafety. After *in-vivo* administration of theranostic nanoparticles to treat gouty-arthritic mice, tissues from the vital organs including the liver, spleen, kidney, lungs, and heart, and the physiologically relevant paw were taken for H&E biosafety and therapeutic efficacy analysis. For this, the tissues were fixed in 10% neutral buffered formalin for 24h, decanted, and stored in 70% ethanol for long term storage at 4°C. The samples were sent to Biospecimen Resource and Molecular Analysis Facility (BRaMA), PVLSI, UMass Chan Medical School-Baystate for histology and analysis. Tissues were embedded in paraffin and sectioned 5 µM thick. The foot and ankle tissues were decalcified before proceeding for embedding to allow smooth sectioning. Sectioned tissues were then stained with hematoxylin & eosin for evaluation of acute toxicity and inflammation in vital organs, and the reduction in inflammation from the treatment groups in the mouse paws. Images were taken at indicated magnification and the histology was examined by the Department of Pathology, UMass Chan Medical School at Bay State Health, Springfield, MA.

Statistical analysis. Statistics were analyzed using GraphPad Prism 8. Two groups comparison analysis was performed by two-tailed unpaired t-test. For comparison of multiple groups, ordinary one-way or two-way ANOVA was utilized followed by Dunnett's or Sidak's multiple comparison test. All the results were represented as mean ± s.e.m. (standard error of the mean) or mean ± s.d. (standard deviation) and p-value less than 0.05 was considered to be significant. For in-vivo experiments, two to five mice were used per experiment, which have been previously shown to be sufficient to calculate statistical significance.

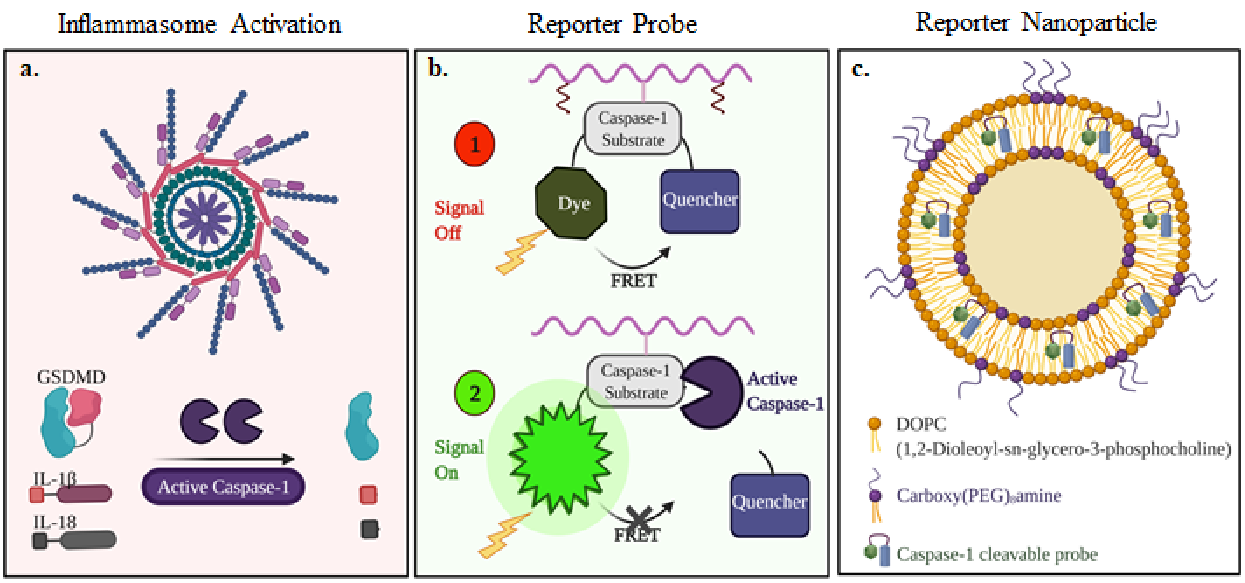

Figure S1. Schematic represents the conceptualization of reporter nanoparticle (FLTD NP) for monitoring of inflammasome activation.

Cy5-KFLTDK(QSY21)-C

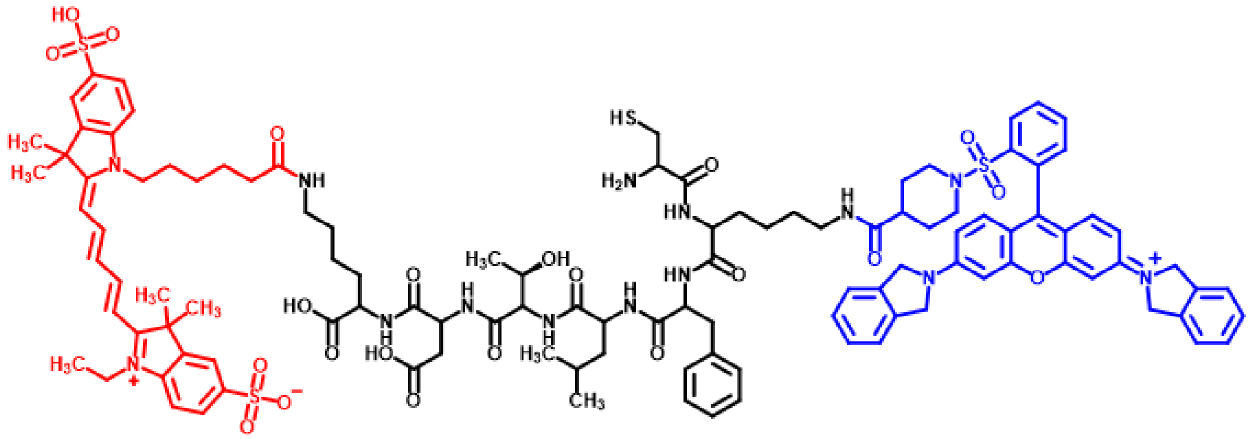

Figure S2. Chemical structure of caspase-1 responsive Cy5-KFLTDK(QSY21)-C probe (in short: -FLTD- probe).

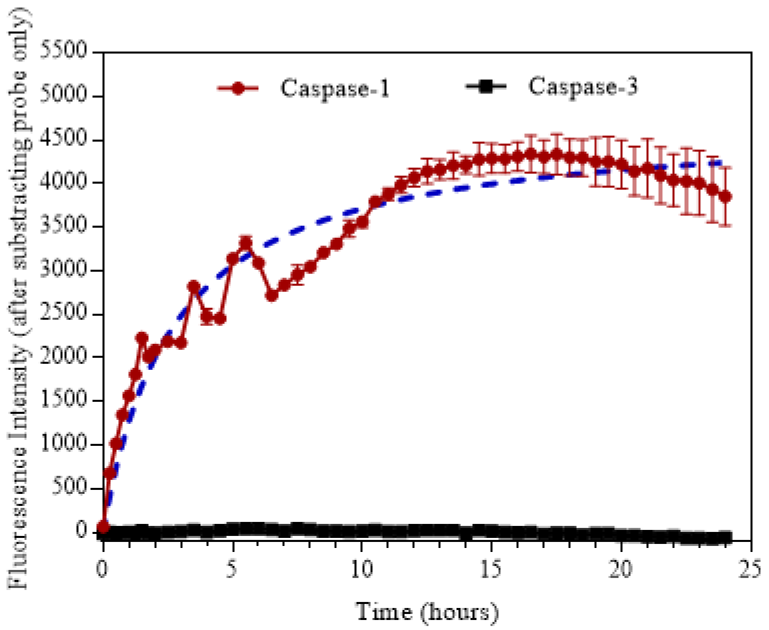

Figure S3: Reporter probe specificity and validation. Graph plots the Cy5 fluorescence intensity emitted upon incubation of reporter Cy5-KFLTDK(QSY21)-C probe with recombinantly active caspase enzymes, caspase-1 and caspase-3. Data shown as mean ± s.e.m. (n=2).

a.

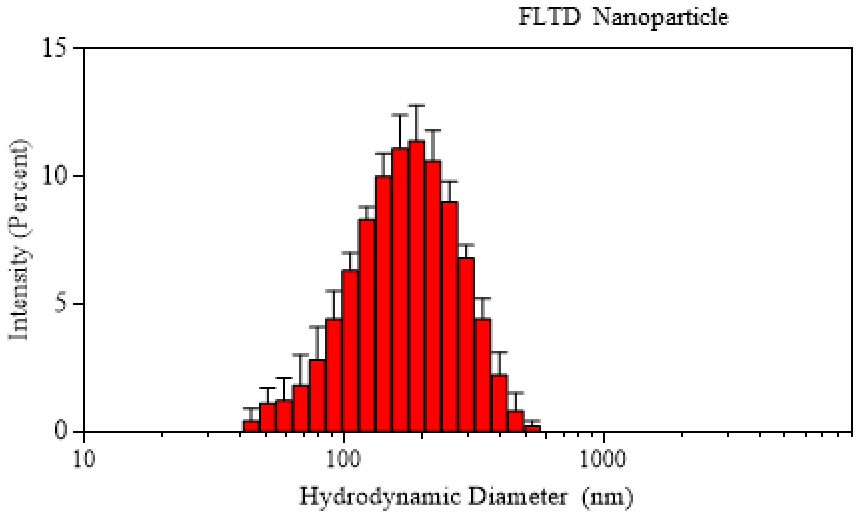

b.

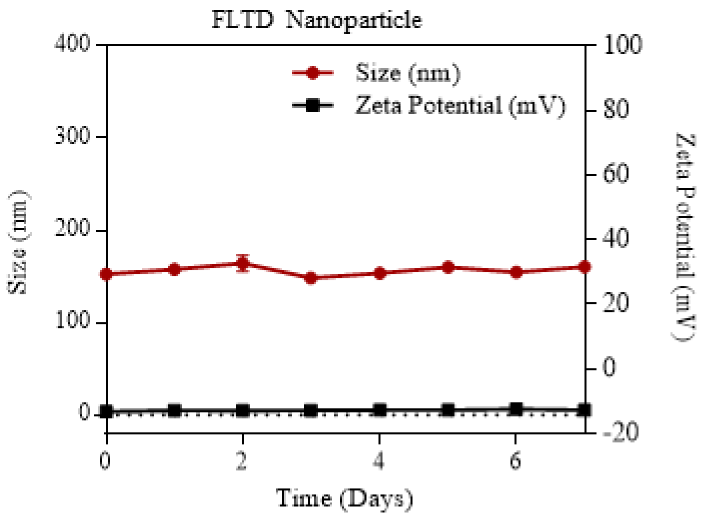

Figure S4: Size and characterization of reporter nanoparticle systems. (a) Graph indicates average hydrodynamic diameter of FLTD NPs obtained from DLS intensity plot. Data in are shown as mean ± s.d. **(b)** Graph represents the PBS stability of FLTD NPs by exhibiting its size and zeta potential in PBS over a course of 7 days. Data shown are mean ± s.e.m. (n=3).

a. b.

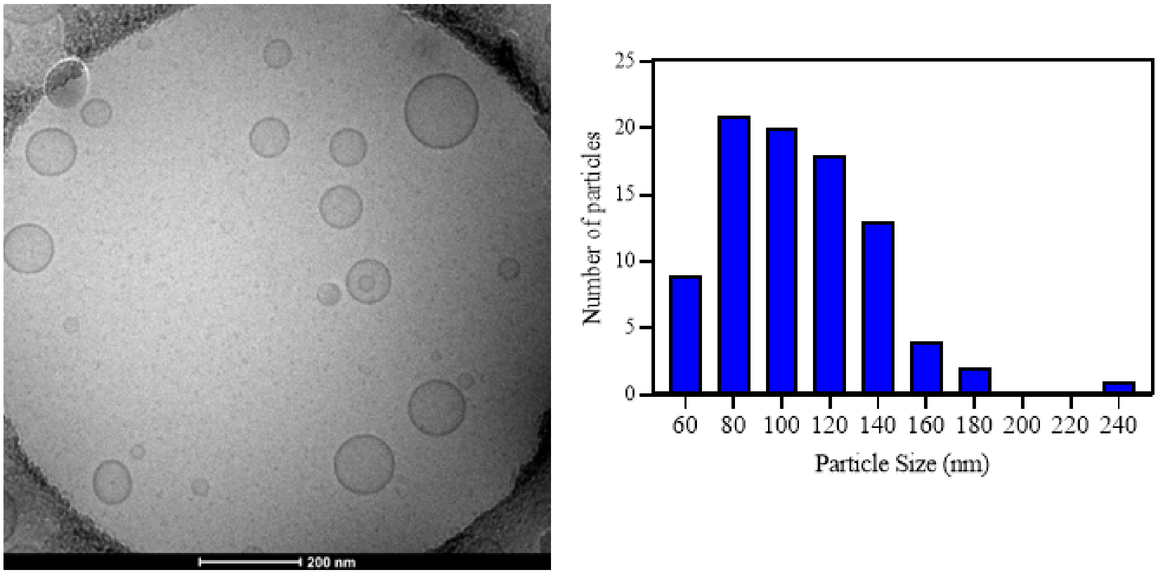

Figure S: Cryo-TEM imaging analysis of FLTD-DSR nanoparticles. (a) Enlarged representative Cryo-TEM image of theranostic FLTD-DSR NP, exhibiting a largely uni-lamellar morphology typical of stable liposomal nanoparticles. **(b)** Size-distribution histogram of TEM images of the nanoparticles, exhibiting an average particle size of 107 ± 31 nm standard deviation. Data represents the mean, non-hydrodynamic size taken from 22 separate TEM images.

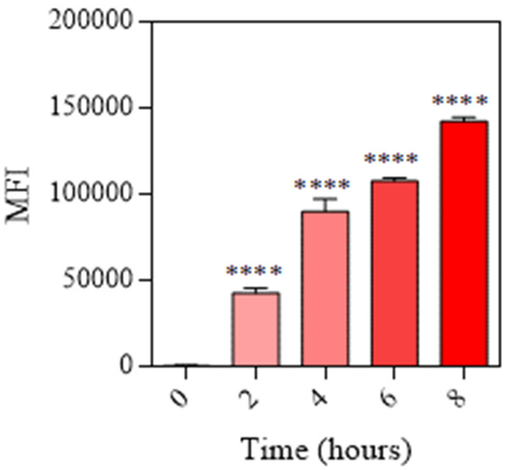

Figure S6- Time-dependent internalization of Cy5 fluorescent nanoparticles. Graphs displays mean fluorescence intensity obtained from internalization of fluorescent nanoparticles by iBMDMs during a time course of 8 hours, acquired by flow cytometry. Data in b, c & d are mean ± s.e.m. (n=3). Statistical analysis was performed by one-way ANOVA and Dunnett's multiple comparisons test. $^{**}p < 0.01$, $^{***}p<0.001$, $^{****}p<0.0001$

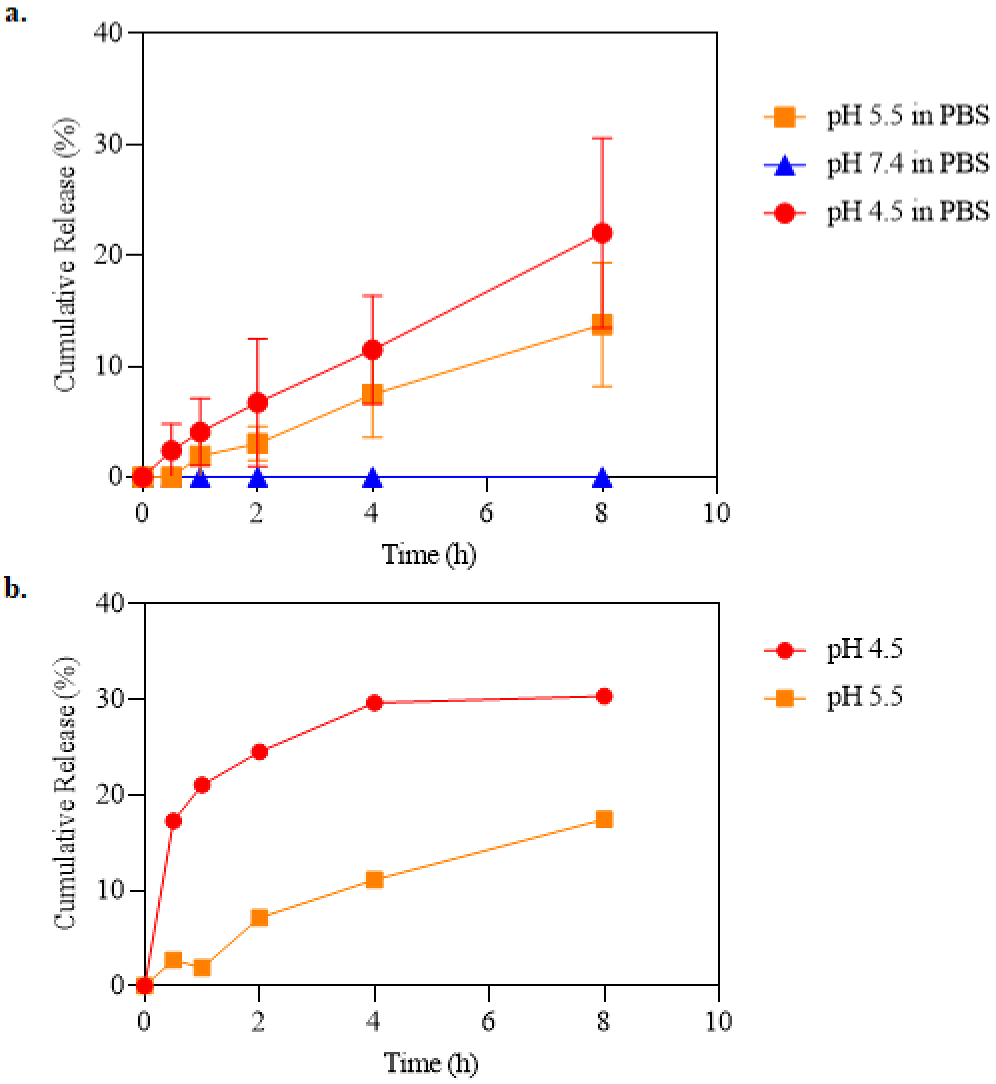

Figure S7: Nanoparticle kinetic breakdown and DiD dye release via dialysis at low pH and in macrophage lysate. Release kinetic study showing the cumulative release of DiD dye from NPs replacing FLTD probe with lipophilic DiD dye at pH 7.4, 5.5, 4.5 in PBS, and at pH 4.5 and 5.5 in macrophage lysate. **(a)** Cumulative release of DiD from NPs in PBS at pH 4.5, 5.5, and 7.4. Data shown is mean ± SEM (n=3). **(b)** Representative cumulative release kinetic study of DiD from NPs incubated in macrophage lysate and dialyzed against pH 4.5 and pH 5.5 PBS.

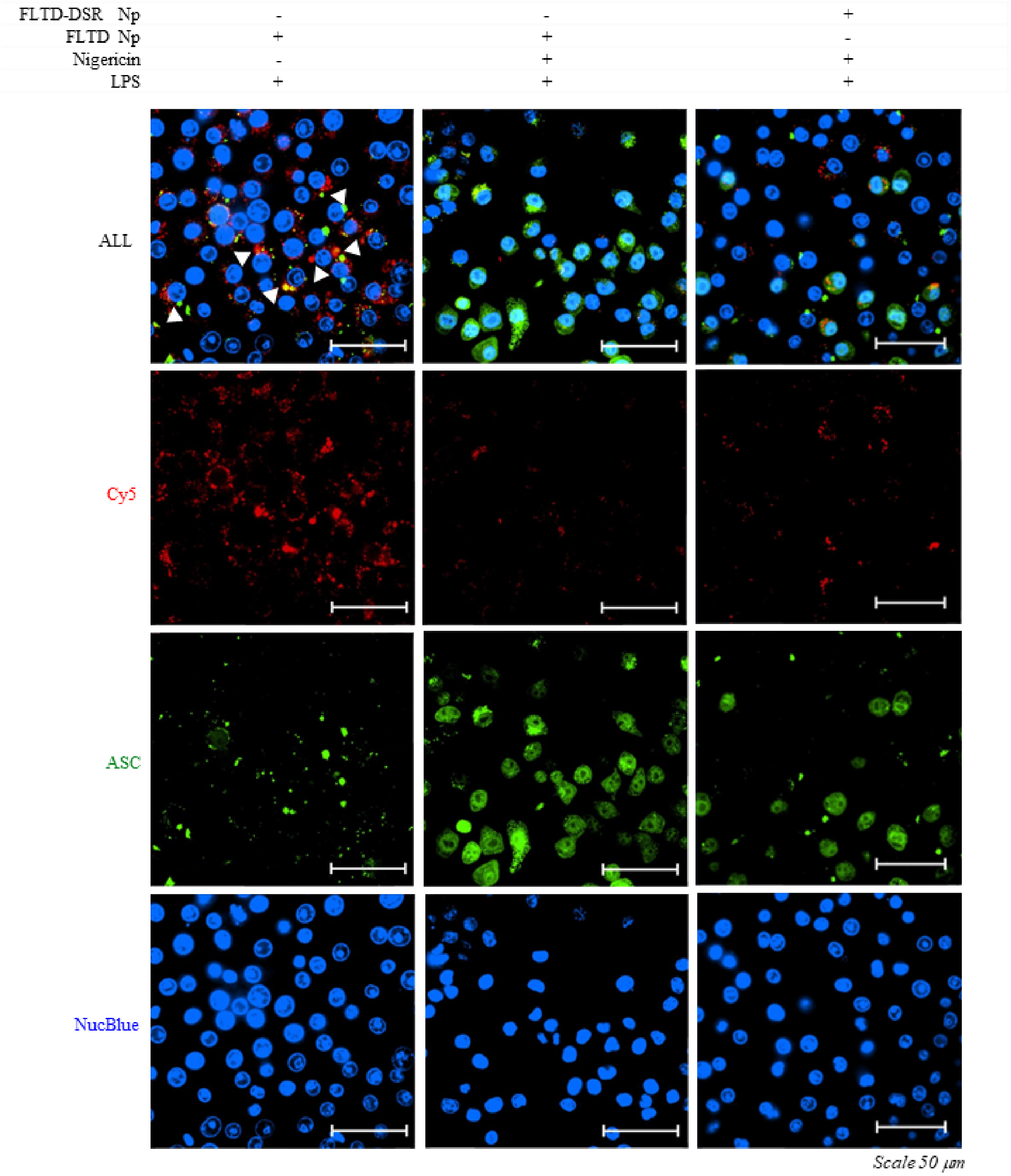

Figure S8: Magnified representative of reporter (FLTD NP) and theranostic (FLTD-DSR NP) nanoparticles *in-vitro* activity test. Representative magnified fluorescent images of only primed and nigericin treated iBMDMs pretreated with reporter FLTD NPs, displaying the activity of the caspase-1 responsive probe. NucBlue: Blue, ASC-Citrine: Green, Cy5 from reporter probe: Red. Scale bar: 50 µm.

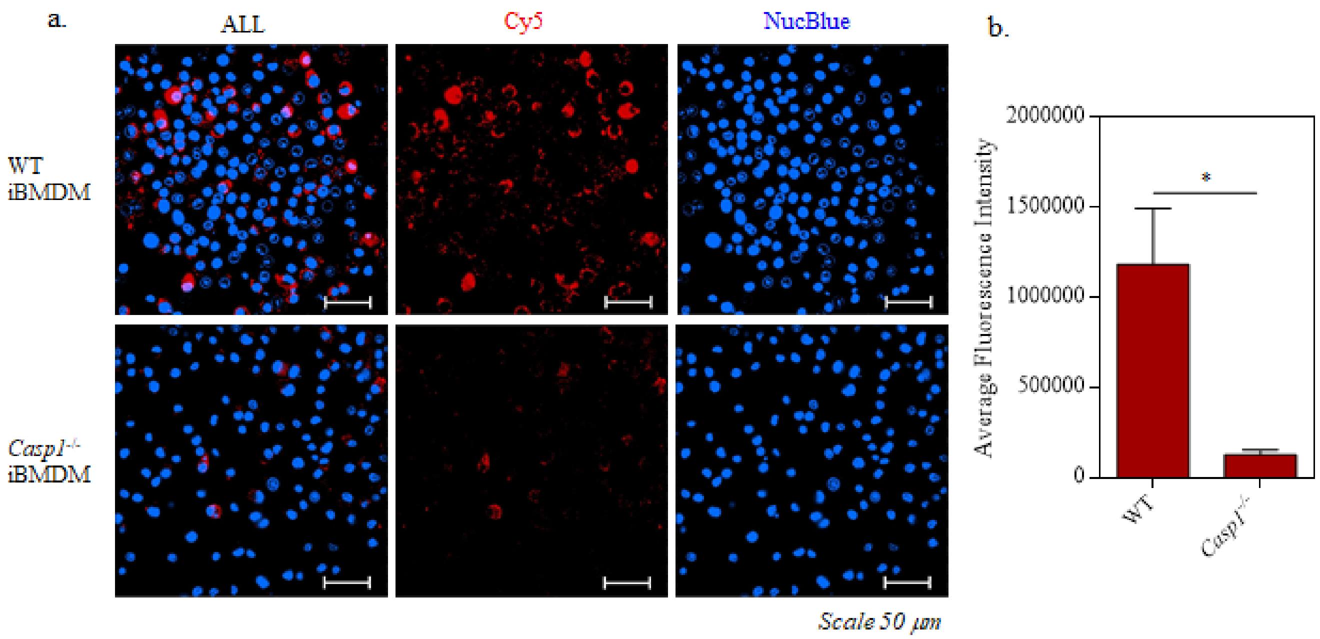

Figure S9: Validation of reporter nanoparticle activity using *Casp1*$^{-/-}$ iBMDM. (a) Representative fluorescent images displaying the activity of FLTD NP in wild-type and *Casp1*$^{-/-}$ iBMDM, to determine the reporter nanoparticle specificity towards caspase-1 activity. Both types of cells were treated with LPS and nigericin in order to activate inflammasome. Images were taken after 30 minutes of nigericin treatment. Cy5: Red, NucBlue: Blue. Scale bar-50 µm. **(b)** Quantitative analysis of Cy5 average fluorescence intensity obtained from reporter nanoparticle in inflammasome activated wild type and *Casp1*$^{-/-}$ iBMDM. Data shows mean ± s.e.m. (n=3). Statistical analysis was conducted using unpaired t-test. *$p < 0.05$

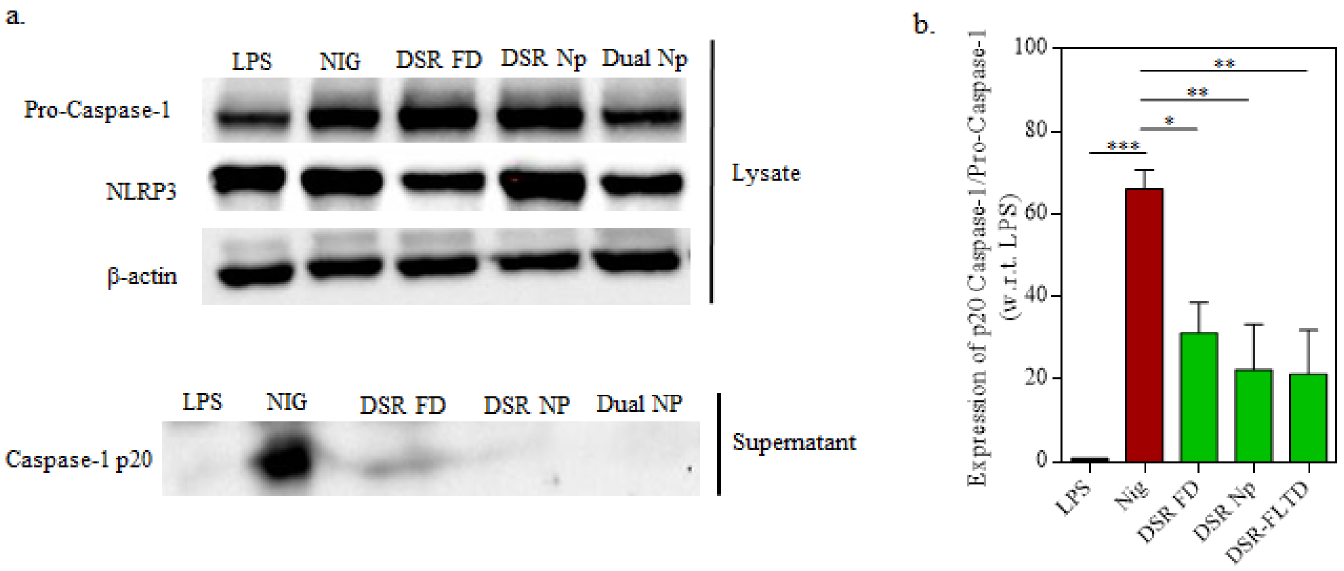

Figure S10: Expression of inflammasome-associated proteins in treated iBMDMs. **(a)** Top panel displays the representative western blot image exhibiting expression of Pro-Caspase-1 and NLRP3 in lysates prepared from free drug and nanoparticle treated iBMDMs. Bottom panel shows the expression of active Caspase-1 (p20) released in the supernatant of these treated cells. **(b)** Quantitative analysis of expression of active Caspase-1 normalized to Pro-Caspase-1, for all the treatments groups with respect to LPS control group. Values shown here are mean ± s.e.m. (n=3). Statistical analysis was performed by one-way ANOVA and Dunnett's multiple comparisons test. *p < 0.05, **p < 0.01, ***p<0.001.

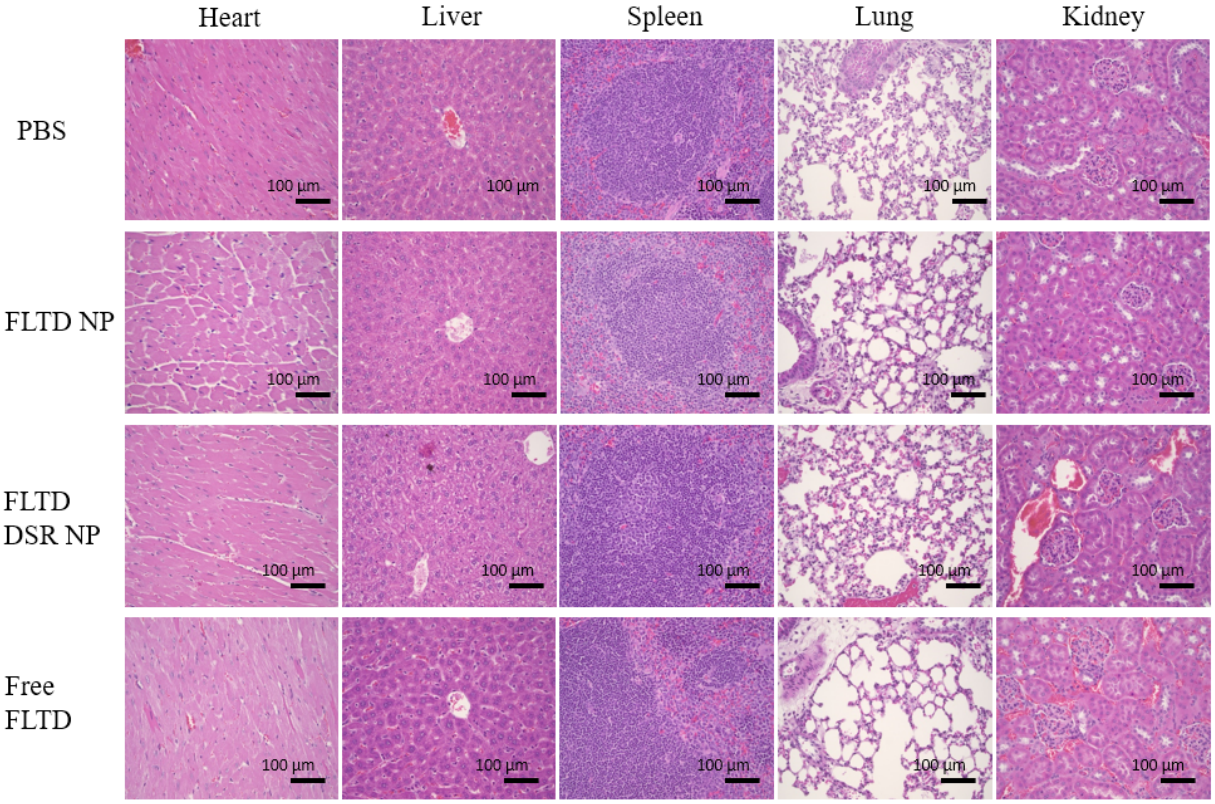

Figure S11: Hematoxylin and Eosin (H&E) staining of mouse organ tissues treated in the gouty arthritis study. H&E stains from heart, liver, spleen, lung, and kidneys of PBS (control), FLTD nanoparticle, FLTD DSR nanoparticle, and free FLTD probe treated mice. Each treatment group exhibits normal histology of the respective organs without any gross abnormalities, indicating that the nanoparticle system is biocompatible and the respective treatments at the subjected concentrations exhibit adequate biosafety and are non-toxic. Scale bar - 100µM.

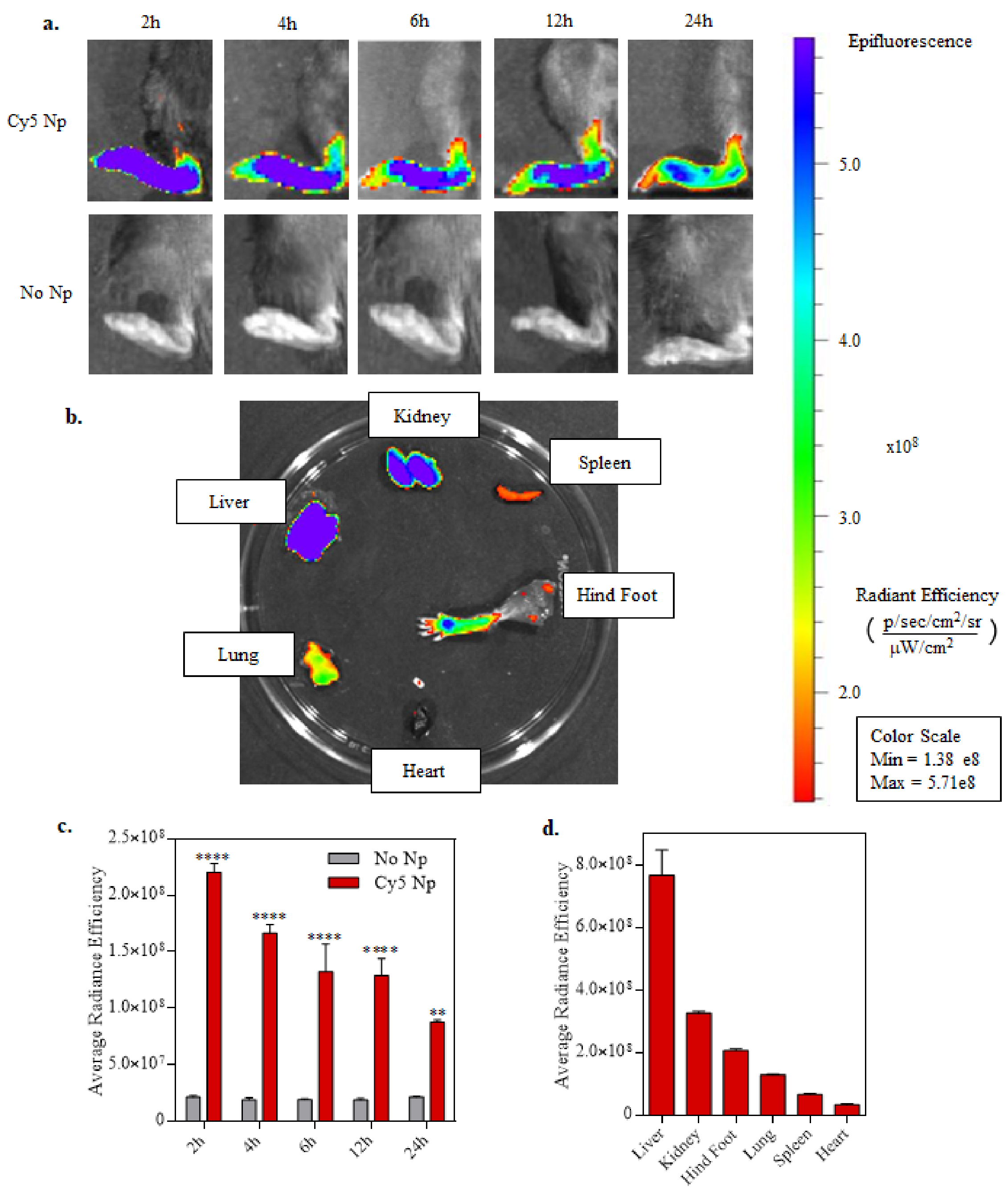

Figure S12: Biodistribution of nanoparticles encapsulated with Cy5-conjugated peptide. **(a)** Representative NiR images displaying accumulation of nanoparticles encapsulated with Cy5-peptide, in MSU-injected inflamed foot over a time course of 24 hours. **(b)** Representative NiR images of organs excised from mice after 24 hours of Cy5 nanoparticle injection. **(c)** Graph plots the average radiance efficiency calculated from NiR signal exhibited in inflamed foot of mice injected with Cy5 nanoparticles or just PBS. Data shown as mean ± s.e.m. (n=2). Statistical analysis was performed by two-way ANOVA and Sidak's multiple comparisons test. $^{**}p < 0.01$, $^{****}p<0.0001$. '*' indicates comparison from no nanoparticle treatment (only PBS) group. d) Graph represents the average radiance obtained from different organs of mice treated with Cy5 nanoparticles for 32 hours. Data shown as mean ± s.e.m. (n=2).

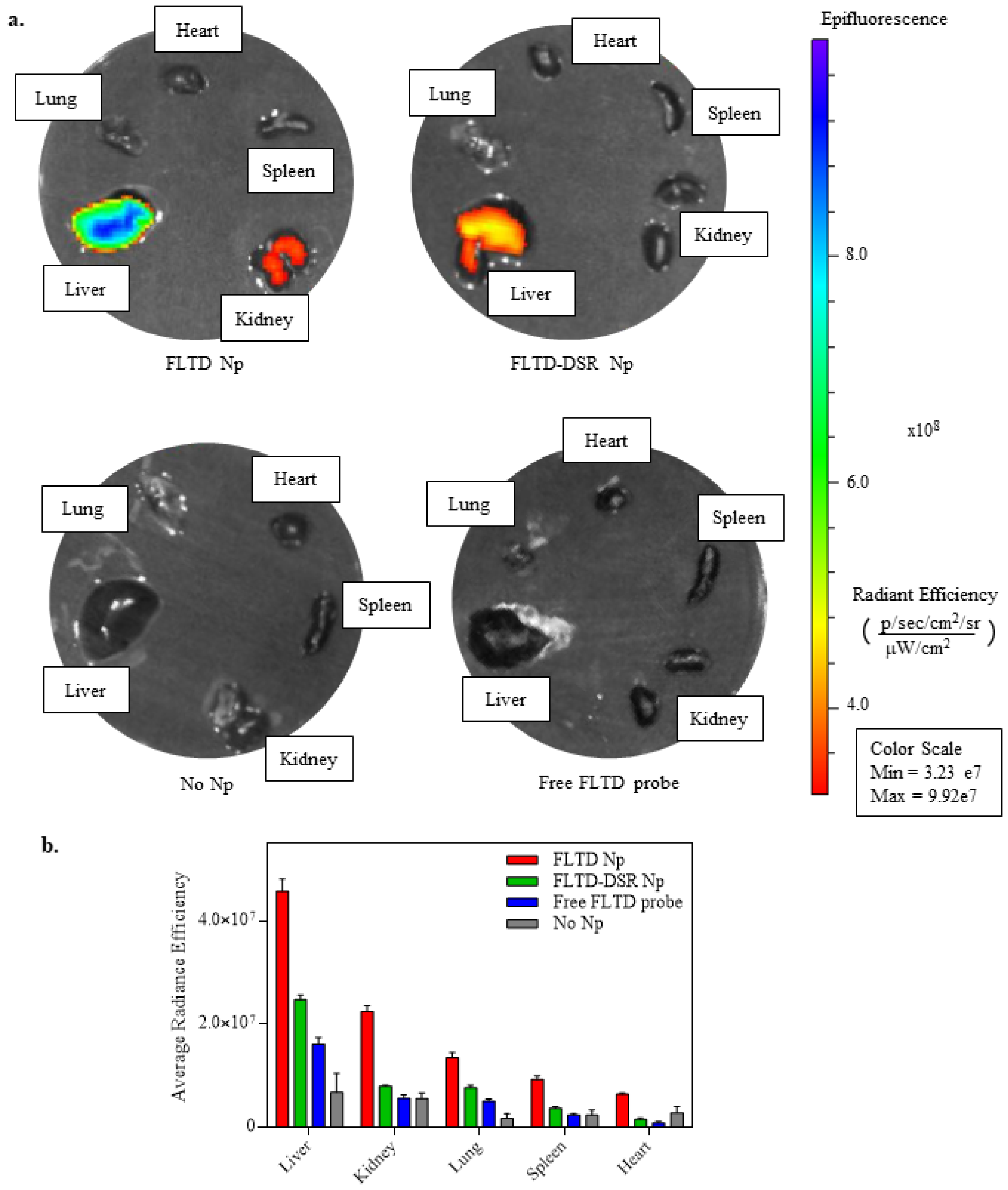

Figure S13: Organ biodistribution of reporter in different treatment groups after 48 hours of injection. (a) Representative NiR images showing the Cy5 signal emitted by organs excised from mice subjected to reporter/theranostic nanoparticles or free probe treatments for 48 hours. **(b)** Quantitative analysis of the reporter signal obtained from different organs of mice after 48 hours of indicated treatments. Data shown as mean ± s.e.m. (n=3).

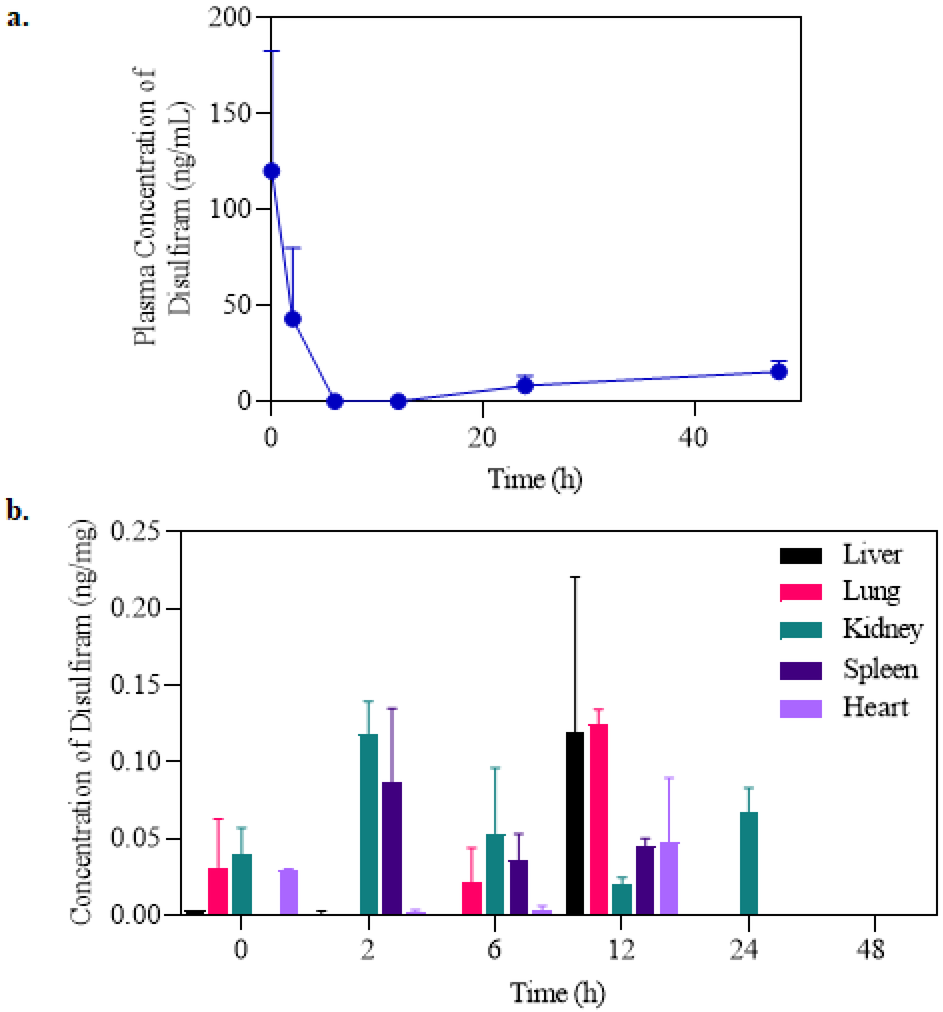

Figure S14: Pharmacokinetic and biodistribution analysis of disulfiram released in mouse tissues by DSR-NPs. (a) Concentration of disulfiram (DSR) detected in the serum of mice injected with DSR-NPs after various time points. Data shown is mean ± SEM (n=2). **(b)** Concentration of DSR detected in the corresponding organs liver, lung, kidney, spleen and heart of mice injected with DSR-NP delivering 10 mg/kg DSR dosage, after various time points. Data shown is mean ± SEM (n=2).

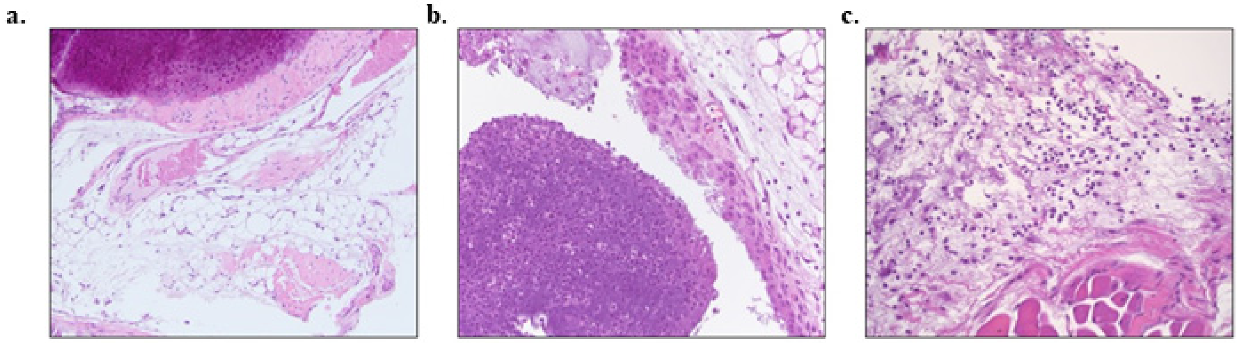

Figure S15: Hematoxylin and Eosin (H&E) staining of MSU-induced, nanoparticle treated gouty arthritis in mouse foot. (a) 40x magnification image of PBS-treated control foot, showing normal skin, subcutaneous tissue, and bone without any inflammatory infiltration. **(b)** 40x magnification of representative images of sections of MSU-induced gouty arthritis joints from mice treated with only probe FLTD nanoparticles, showing extensive necrotizing inflammatory infiltration composed of neutrophils, lymphocytes, and plasma cells, with histiocytes in the periphery of the necrotized area. **(c)** 40x magnification images of sections of MSU-induced gouty arthritis induced mice treated with FLTD-DSR nanoparticles, showing a resolution of inflammation. Extensive dermal and subcutaneous edema is noted with sparse inflammatory infiltrates, predominantly composed of lymphocytes and plasma cells.

References

[1] P. Sengupta, S. Basu, S. Soni, A. Pandey, B. Roy, M. S. Oh, K. T. Chin, A. S. Paraskar, S. Sarangi, Y. Connor, V. S. Sabbisetti, J. Kopparam, A. Kulkarni, K. Muto, C. Amarasiriwardena, I. Jayawardene, N. Lupoli, D. M. Dinulescu, J. V. Bonventre, R. A. Mashelkar, S. Sengupta, **2012**.
[2] S. M. Roberson, W. S. Walker, *Cell Immunol* **1988**, 116, 341.
[3] D. De Nardo, D. Kalvakolanu, E. Latz, *Macrophages. Methods in Molecular Biology* **2018**, 1784, 35.
